# “Digital Clinicians” Performing Obesity Medication Self-Injection Education: Feasibility Randomized Controlled Trial

**DOI:** 10.2196/63503

**Published:** 2025-07-30

**Authors:** Sean Coleman, Caitríona Lynch, Hemendra Worlikar, Emily Kelly, Kate Loveys, Andrew J Simpkin, Jane C Walsh, Elizabeth Broadbent, Francis M Finucane, Derek O' Keeffe

**Affiliations:** 1 Department of Diabetes, Endocrinology, and Metabolism, University Hospital Galway, Newcastle RdGalway, H91 YR71, Ireland, 353 851604880; 2 School of Medicine, University of Galway, Galway, Ireland; 3 School of Psychology, University of Auckland, Auckland, New Zealand; 4 School of Mathematics and Statistical Science, University of Galway, Galway, Ireland; 5 School of Psychology, University of Galway, Galway, Ireland

**Keywords:** automation, automate, machine-human interface, clinical education, digital clinician, virtual human, trust, chatGPT, chatbots, machine learning, ML, artificial intelligence, AI, large language models, natural language processing, NLP, deep learning, randomized controlled trials, RCTs, feasibility studies, obesity, medication

## Abstract

**Background:**

Artificial intelligence (AI) chatbots have shown competency in a range of areas, including clinical note taking, diagnosis, research, and emotional support. An obesity epidemic, alongside a growth in novel injectable pharmacological solutions, has put a strain on limited resources.

**Objective:**

This study aimed to investigate the use of a chatbot integrated with a digital avatar to create a “digital clinician.” This was used to provide mandatory patient education for those beginning semaglutide once-weekly self-administered injections for the treatment of overweight and obesity at a national center.

**Methods:**

A “digital clinician” with facial and vocal recognition technology was generated with a bespoke 10- to 15-minute clinician-validated tutorial. A feasibility randomized controlled noninferiority trial compared knowledge test scores, self-efficacy, consultation satisfaction, and trust levels between those using the AI-powered clinician avatar onsite and those receiving conventional semaglutide education from nursing staff. Attitudes were recorded immediately after the intervention and again at 2 weeks after the education session.

**Results:**

A total of 43 participants were recruited, 27 to the intervention group and 16 to the control group. Patients in the “digital clinician” group were significantly more knowledgeable postconsultation (median 10, IQR 10‐11 vs median 8, IQR 7‐9.3; *P*<.001). Patients in the control group were more satisfied with their consultation (median 7, IQR 6‐7 vs median 7, IQR 7‐7; *P*<.001) and had more trust in their education provider (median 7, IQR 4.8‐7 vs median 7, IQR 7‐7; *P*<.001). There was no significant difference in reported levels of self-efficacy (*P*=.57). 81% (22/27) participants in the intervention group said they would use the resource in their own time.

**Conclusions:**

Bespoke AI chatbots integrated with digital avatars to create a “digital clinician” may perform health care education in a clinical environment. They can ensure higher levels of knowledge transfer yet are not as trusted as their human counterparts. “Digital clinicians” may have the potential to aid the redistribution of resources, alleviating pressure on bariatric services and health care systems, the extent to which remains to be determined in future studies.

## Introduction

Obesity, an “abnormal or excessive accumulation of fat that poses a health risk” [1], is a primary contributor to global health challenges [2]. Reports suggest its involvement in up to 80% of cases of type 2 diabetes and 43% of cardiovascular incidents [3] while significantly contributing to depression and anxiety [4].

Recent breakthroughs in bariatric medicine have elevated the role of injectable therapy, namely glucagon-like peptide-1 agonist agents such as semaglutide. New injectable agents are showing weight loss surpassing 20% and 25% [5]. With over half of adults in the World Health Organization (WHO) European Region [2] potentially eligible, use is hindered by scalability of clinical services and supply. Generative AI has been identified to have the potential to offset the clinical and administrative demands associated with the management of patients on these medication types [6]

Patient education is a critical component of the clinical care pathway and a prerequisite at many clinics for the prescription of pharmacotherapy. Even in resource-rich countries, the necessary services are not always available. Studies have predicted the United States needs careful restructuring of health service expenditure to meet demand for the costs of overweight and obesity services [7].

Health care systems are underresourced and understaffed, particularly in rural areas [8]. This impacts productivity, sustainability [9], and the health of both patient and health care professionals [10]. Health care worker shortages, propelled by aging populations [11] and the COVID-19 pandemic [12], are exacerbated in areas such as the Global South, increasing health care inequities [13]. Tasks that health care workers perform are often repetitive and administrative. Redistribution of such tasks has been shown to potentially improve health outcomes such as blood pressure, HbA_1c_, and mental health [14].

Artificial Intelligence (AI) chatbots have the potential to be used for a variety of medical tasks, including note taking [15] and personalized medicine [16]. Anonymized AI chatbots have been judged to provide better, more concise, empathetic answers to general health queries than verified physicians [17]. ChatGPT-3 is known to be accurate with common chief complaints [18] and GPT-4 recently outscored 99.98% of simulated human readers when diagnosing complex clinical cases [19]. Automated personalized messaging systems are being researched to enhance behavioral change in a hope to enhance health outcomes [20].

However, there are concerns about trust, usability, and efficacy [21]. Hallucinating models can spread misinformation and private medical data may be misused [16]. Trust is fundamental to the physician-patient relationship shown to affect health behaviors, compliance, and quality of life [22]. Willingness to use supportive technologies has been shown to be influenced by complex factors, such as perceived usefulness, health threat, and resistance to change [23]. Chatbots are text-based and have rarely been integrated with physical form, for example, avatars or “virtual humans.” Adding form to a faceless chatbot to create a “digital clinician” may increase trust, engagement, and usability [24].

Chatbots are text-based and have rarely been integrated with physical form, for example, avatars or “virtual humans.” Adding form to a faceless chatbot to create a “digital clinician” may increase trust, engagement, and usability [24].

Automation may offer benefits in standardization, efficiency, effectiveness, cost, confidentiality, and access. Social desirability response bias is associated with higher levels of treatment nonadherence [25] and reduces the accuracy of clinical history taking [26] in human-human interactions. In educational settings, certain interactions, such as quizzing, enhance information retention but may be more socially appropriate from a “digital clinician” than from a health care professional.

Virtual humans have been shown to be efficacious in nonclinical patient-facing scenarios [27]. There has been a paradigm shift since the advent of ChatGPT, bringing automated communicators into a new light, mandating research focus. The aim of this study is to investigate the use of a medically approved task-specific “digital clinician” to provide patient education in a clinical environment, comparing it with a human counterpart.

## Methods

### Research Design

All patients at Galway University Hospitals must attend an education session with a Clinical Nurse Specialist before starting semaglutide injectable therapy. This session covers basic semaglutide pharmacotherapy and the safe self-administration of injections using a semaglutide pen. During the study period, allocated, eligible participants received their mandatory education from a “digital clinician” with human oversight. The control group received current standard-of-care, human, nurse-led education.

Once a clinical decision was made that treatment of overweight or obesity with semaglutide injections would be commenced, eligibility was assessed. Overweight and obesity were classified as a BMI greater than 25 kg/m^2^. Eligibility criteria included being aged 18 years or older, without an intellectual or physical disability, which would interfere with a participant’s ability to self-administer semaglutide injections. All eligible patients were invited to participate in the study. Given the nature of the intervention (“digital clinician”), blinding was not feasible.

Eligibility criteria included patients aged older than 18 years, without an intellectual or physical disability, which would interfere with a participant’s ability to self-administer semaglutide injections. All eligible patients were invited to participate in the study. Given the nature of the intervention (“digital clinician”), blinding was not feasible.

### The “Digital Clinician”

To inform design, clinicians were observed educating patients. Their mannerisms, behavior, and conversations were recorded. Transcripts of educational sessions given by the local obesity nurse specialist were generated.

A 10- to 15-minute educational script was generated with multiple conversation streams. Information on medication name, dosages, mechanism of action, pen preparation, administration, side effects, and storage was included. User questioning was used to maximize engagement and knowledge retention.

Akin to natural clinical education, the “digital clinician” led the primary section of the tutorial, offering information and asking the patient questions throughout to test retention and assess understanding. The question types used by the “digital clinician” varied. Some were open, while others were multiple choice, true or false, yes or no, or numerical. After the clinician-led portion of the tutorial had ended, patients were invited to type or voice any question they wished. The option to see and hear the answers to some frequently asked questions was also offered by way of onscreen prompts.

Unclear or ambiguous responses were identified by the digital clinician as such, and this was communicated to the user who was then kindly asked to repeat themselves. If not understanding the user twice in a row, the “digital clinician” would simply offer the correct answer and continue. This prevented the possibility of an infinite loop. When offered the chance to ask general queries, the user may query infinitely if they wish, although a comprehensive list of frequently asked question prompts on screen, and an onscreen button to end the tutorial, were in place to reduce this need.

A careful selection of trigger words and combinations of “IF,” “AND,” “OR,” and “NOT” statements was used to design the conversational flow. Rigorous internal testing, including over 100 iterations of the conversational flow, was tested by the research team to ensure a fluent, cohesive, usable digital clinician with little risk of misinterpreting the user’s intent.

This “digital clinician” could be accessed with native web browsers on the participants’ phone, tablet, or laptop. In this case, the participants accessed the session under supervision on an iPad (Apple) device.

Two multiscene videos were recorded by the team and integrated into the tutorial using screen-in-screen projection. The videos offered human views and instructions of what preparing and using the injection pen entailed. The videos, while being demonstrated by members of the research team, were narrated by the “digital clinician.”

The software IBM Watson Assistant was used to generate conversation streams. Microsoft ClipChamp Video Editor was used to edit videos, then uploaded to Imgur, accessed via source code written on IBM Watson. The IBM Watson conversation code file was integrated with an animated avatar on the SoulMachines Creator Website. The “digital clinician” uses natural language processing and an evolving bank of AI-driven animation responses that monitor vocal tone and facial expression to regulate behavior. The “digital clinician” has a programmed personality, name, voice, and identity and is being used in a role traditionally considered to require human intelligence.

Logos of affiliated educational and health care institutions were included on the user interface screen to increase trust. Cinematic screening offered different angles of the avatar's face to make for a more interesting experience. Multiple avatars of different genders, races, and names were used to promote inclusivity. All possible conversation streams had been verified against official pharmaceutical literature. The individual scripts were not recorded, but each participant-AI interaction was supervised to monitor for technical issues or hallucinations. Multiple avatars of different genders, races, and names were used to promote inclusivity. All possible conversation streams had been verified against official pharmaceutical literature. The individual scripts were not recorded, but each participant-AI interaction was supervised to monitor for technical issues or hallucinations. A short video of the “digital clinician” in use is included in Multimedia Appendix 1

### Randomization: Minimization

Minimization is a method used in clinical trials and experimental research to allocate participants [4,28]. Participants were allocated in order of enrollment to the study arm that minimized the difference across 4 selected numerical baseline variables, namely, age, BMI, pretutorial knowledge of semaglutide score, and pretutorial injection self-efficacy score. Order of enrollment in the study was determined by check-in time at the clinic. Allocation by minimization is deterministic and hence precludes traditional concealment.

The first participant was assigned at random using a coin toss. The second participant was subsequently assigned to the alternative group. Allocation was verified using Microsoft Excel by an independent assessor with access to study data in real-time at a separate location via a secure web-based suppository. The assessor then communicated the allocation to the research team on site.

Allocation was verified using Microsoft Excel by an independent assessor with access to study data in real-time at a separate location via a secure online suppository. The assessor then communicated the allocation to the research team on site.

### Self-Efficacy

Self-efficacy was measured using the 2-part validated Self Injection Assessment Questionnaire (SIAQ) [29]. The preinjection SIAQ was used as baseline data. The postinjection SIAQ was scored across 5 domains considering 2-week postintervention feelings about injections, self-image, self-confidence, pain and skin reactions, ease of use, and satisfaction. The SIAQ has been shown to be a valid, robust tool with sufficient validity, reliability, consistency, and sensitivity. Cronbach α and the test-retest coefficient were >0.70 for all domains [30].

### Knowledge Attainment

Unvalidated knowledge assessments were designed specifically for the purpose of this research. The pre-educational assessment tool consisted of 4 questions. The posteducational assessment tool contained the same initial 4 questions and a further 8 questions. Questions were multiple choice and covered topics, such as drug name, drug class, mechanism of action, side effects, injection technique, and storage requirements.

### Consultation Satisfaction

The validated Patients’ Overall Satisfaction with Primary Care Physicians Scale (CPSS) [31] has criterion-related validity coefficients mostly in the 0.80s and 0.90s when considering empathy, physician recommendation, and general satisfaction. Cronbach coefficient α for the patient satisfaction scale is 0.98. The 7-point Likert scale was used in the study.

### Trust

The “Trust between People and Automation Scale” (TPA) has undergone validation [32] and been in use for more than 20 years. It was adapted and integrated with the consultation satisfaction measure for this study.

### Technology Usability and Future Use

The validated Technology Usability Questionnaire (TUQ) [33] was adapted. The questionnaire has high reliability with Cronbach Coefficient α more than 0.8 across all domains, and high validity expressed through its multiple native questionnaires [34]. A shortened version including questions from 4 of 5 domains was used. The domains were “Ease of use and Learnability,” “Interface Quality,” “Interaction Quality,” and “Satisfaction and future use.” Open-ended questions were used to record general themes and attitudes toward the “digital clinician.” The outcome measure tools are included as Multimedia Appendix 2.

### Sample Size Calculations

When calculating sample size, a similar study using the validated SIAQ to measure self-efficacy was used [29]. It showed a mean 7.09 (SD of 1.5), on a 10-point Likert scale. A significant difference of two-thirds the SD was proposed to boundary noninferiority, with 80% power and α=.05; this would require 28 participants to be recruited into each arm of the study.

### Ethical Considerations

A randomized controlled noninferiority trial of “digital clinician”-led patient education was designed with ethical approval from Galway University Hospitals, Clinical Research Ethics Committee (Ref: CA 2920). The trial and trial protocol were guided by SPIRIT-AI (Standard Protocol Items: Recommendations for Interventional Trials involving Artificial Intelligence) [35] and CONSORT-AI (Consolidated Standards of Reporting Trials- Artificial Intelligence) [34] extensions. Further information on the study protocol can be found in Multimedia Appendix 3. It was ensured that all patients were offered at least the current standard of care. After data collection, all patients were seen by the clinical nurse specialist. The nurse assessed the patients who had received safety-net instructions before being discharged with a medication information leaflet. Contact with the nurse minimized disruption of the patient-clinician relationship. This established human oversight and accountability. Informed written consent was obtained from all participants before enrollment and randomization, after provision of a patient information leaflet and discussion with the research team. All patient data was anonymized. No patient identifiable information was included in data analysis and confidentiality was maintained. Data was stored on password-protected encrypted devices. No compensation was offered to participants.

### Statistical Analysis and Missing Data

Data were analyzed using Jamovi version 2.4.14 developed by Love, Droppman, and Selker [36] and R Studio, developed by Posit PBC [37]. All Likert scale data were appropriately aggregated in treatment and control arms and recalibrated to scales from 1 to 10 for self-efficacy, and 1-7 for trust, satisfaction, and usability.

The Mann-Whitney U test was used to assess differences in primary and secondary outcome variables. This nonparametric test is robust to the distribution of the outcome data. The Mann-Whitney *U* test compares the ranks of all the data points in 2 groups. *P* values below .05 were deemed statistically significant. Conventional content analysis was used to interpret qualitative data. Missing outcome data were omitted from analysis. Observed outcome data were analyzed as if representative of the entire cohort.

Knowledge, satisfaction, trust, and usability were measured immediately posttutorial. Subsequent loss to follow-up at 2 weeks did not affect the handling of this data.

Those lost to follow-up were unavailable for self-efficacy analysis. The profile of those with complete data and those with missing data were explored in Multimedia Appendix 4.

## Results

### Baseline Characteristics

**Figure 1.**
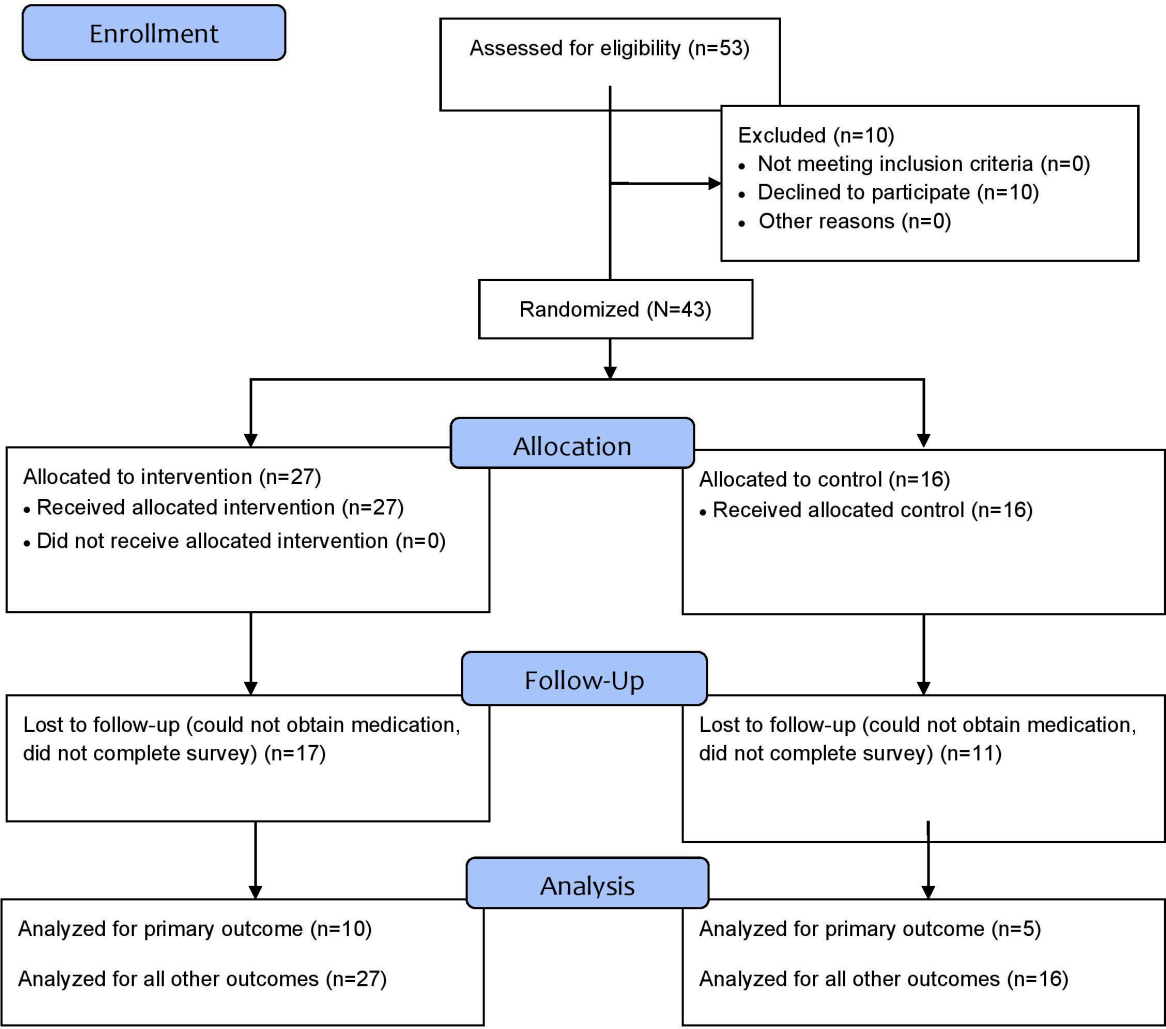
A flowchart of enrollment, allocation, and attrition details.

During the 7-week study period in the bariatric clinic, 53 patients were prescribed semaglutide self-administered injections for the treatment of overweight or obesity (see Figure 1). Of this cohort, 43 agreed to participate in the study. Of those enrolled, 100% (43/43) completed the pretutorial questionnaire, their assigned education session, and their posttutorial questionnaire. Only 41.9% (18) participants completed the 2-week follow-up self-efficacy questionnaire (Table 1).

**Table 1.** A summary of participants' baseline characteristics.

Characteristic		Total (n=43)	Digital clinician (n=27)	Control (n=16)	*P* value
Age, mean (SD)		47.41 (13)	46.4 (13)	49.2 (13)	—
BMI, mean (SD)		43.6 (8)	42.9 (6.8)	44.8 (10)	—
Sex, n (%)					
Male		9 (21)	3 (11)	6 (38)	—
Female		34 (79)	24 (89)	10 (63)	—
Ethnicity, n (%)					
Irish		30 (70)	19 (70)	11 (69)	—
Other		4 (9)	3 (11)	1 (6)	—
Missing		9 (21)	5 (19)	4 (25)	—
Education level, n (%)					
None		1 (2)	1 (4)	0 (0)	—
Primary		2 (5)	1 (4)	1 (6)	—
Secondary		19 (44)	12 (44)	7 (44)	—
Third level		21 (49)	13 (48)	8 (50)	—
Pretutorial Knowledge, median (IQR)		2 (1‐2)	2 (1‐3)	2 (1‐2)	.41
Pretutorial Self Efficacy, median (IQR)		27 (31‐33)	32 (27‐33)	31 (27.8‐33.3)	1.00

### Self Efficacy

**Figure 2.**
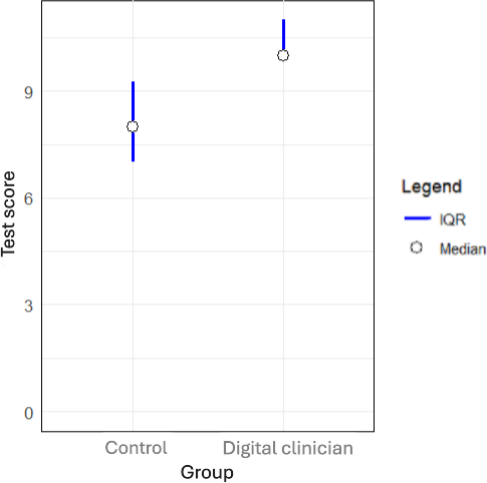
Self-Efficacy scores across control and digital clinician groups. SIAQ: Self-Injection Assessment Questionnaire.

Both groups had a median self-efficacy level of 10/10, with the “digital clinician” group having a marginally lower first quartile of 8, compared with the control group (8.2; Figure 2). This difference was not statistically significant (Table 2; *P*=.52).

Responses recording “Pain and Skin reactions” from the control group had a favorable, nonsignificantly higher mean rank.

**Table 2.** Self-injection self-efficacy assessment results at 2 weeks post intervention.

Domain	Digital clinician (n=10), median (IQR)	Control (n=5), median (IQR)	*P* value
Feelings about injections	5.0 (5.0-5.0)	5.0 (4.0-5.0)	.08
Self-image	5.0 (4.0-5.0)	5.0 (5.0-5.0)	.66
Self-confidence	4.5 (4.0-5.0)	5.0 (4.0-5.0)	.45
Pain and skin reactions	5.0 (5.0-5.0)	5.0 (5.0-5.0)	.08
Ease of use	6.0 (5.0-6.0)	5.0 (5.0-6.0)	.22
Satisfaction	5.0 (4.0-5.0)	5.0 (4.0-5.0)	.61
Overall self-efficacy	10.0 (8.0-10.0)	10.0 (8.2-0.0)	.52

### Knowledge (Test Score)

**Figure 3.**
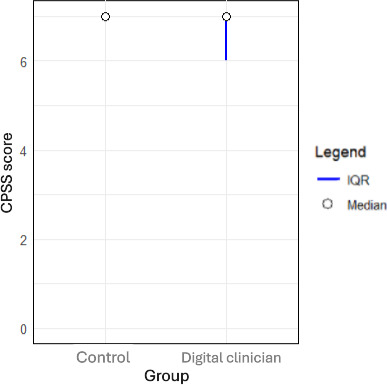
Knowledge scores across control and digital clinician groups. SIAQ: Self Injection Assessment Questionnaire.

Overall, the median test score of participants postconsultation in the “digital clinician” group was 10/12 (84%; IQR 10-11), while the median score in the control group was 8/12 (69%; IQR 7-9*; P*<.001; Figure 3, Table 3).

**Table 3.** Knowledge, trust, and satisfaction levels were assessed in participants that received their education from both the "digital clinician" and the clinical nurse specialist (controls).

Secondary Outcome	Digital clinician (n=27), median (IQR)	Control (n=16), median (IQR)	*P* value
Knowledge	10.0 (10.0-11.0)	8.0 (7.0-9.3)	<.001
Trust-Distrust Scale Domain			
I am confident of the nurse’s knowledge and skills	7.0 (7.0-7.0)	7.0 (7.0-7.0)	.18
The nurse cares about you as a person	6.0 (4.0-7.0)	7.0 (7.0-7.0)	.003
I am (not) suspicious of the nurse’s intentions and actions	5.0 (1.5-7.0)	7.0 (6.0-7.0)	.002
Overall Trust	7.0 (4.8-7.0)	7.0 (7.0-7.0)	<.001
Consultation Satisfaction			
Overall Satisfaction	7.0 (6.0-7.0)	7.0 (7.0-7.0)	<.001

### Consultation Satisfaction

**Figure 4.**
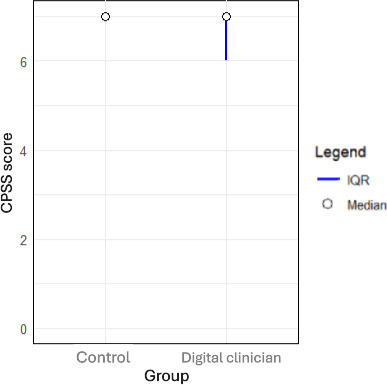
Satisfaction scores across control and digital clinician groups. CPSS: Overall Satisfaction with Primary Care Physicians Scale

In total, 44% (12/27) respondents in the “digital clinician” group were more than 90% satisfied. The lowest score was 63%. In the control group, 94% (15/16) respondents were more than 90% satisfied with their consultation. The lowest score was 86%. The IQR of the “digital clinician group” was 6-7 (median 7), whereas it was 7-7 (median 7) in the control group (Mann-Whitney *P*<.001). This is shown in Figure 4 and Table 3 on a Likert scale between 1 and 7.

### Trust

**Figure 5.**
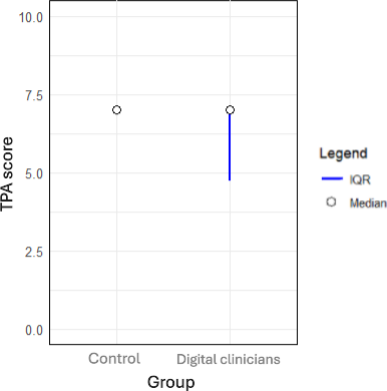
Trust scores across control and digital clinician groups. TPA: Trust between People and Automation Scale.

Figure 5 shows that participants in the “digital clinician” group scored their education provider lower for empathy (*P*=.003), clear intentions (*P*=.002), and overall trust (*P*<.001).

While Figures4  5 show that the medians of the ordinal data are the same, the Mann Whitney U test is nonparametric and is not dependent on central tendency. As nonparametric data can be asymmetrical by nature, central tendency can be misleading. While the medians are the same, the IQR and ranks of the groups are shown to be significantly different with a *P* value <.05.

### Resource Quality

On a scale of 100, the “digital clinician” was found to be 86.667 usable (Table 4), scoring lowest for interface quality and highest for interaction quality.

Of the 27 participants in the intervention group, 22 (81%) of participants affirmed they would use the “digital clinician” for educational purposes in their own time, while 2 (7%) of participants recorded that “maybe” they would. A total of 3 (11%) said they would not.

**Table 4.** The Usability of the "digital clinician" was rated by the user across three domains, scoring highest for interaction quality and ease of use, with 82% of users reporting they would use the resource in their own time.

Usability Domain	Values, mean (SD)
Interaction quality	89.6 (15.1)
Interface quality	81.5 (17.4)
Ease of use	88.9 (16.0)
Overall	86.7 (16.4)
Would you use this resource in your own time? n (%)	
Yes	22 (82)
No	3 (11)
Maybe	2 (7)

### Analysis of Open-Ended Feedback

Below are the described questions about the analysis of open-ended feedback.

*Question: “What do you think about using avatars with automated conversation in a healthcare environment?”*

Four responses made reference to a great invention or great possibility for future use. Seven said the idea was good, or very good. Four described it as “okay.” One respondent felt the avatar was “not ideal,” and another stated they would “prefer a human.” Two responses were of mixed sentiment:

“*I don’t think the real nurse can be replaced but it is great tool for people who live far away to refer to”… “They are helpful but. They are not as good as having a human explaining things to you*.”

*Question: “What concerns would you have about using avatars with automated conversation like this in the future?”*

Twelve responded that they would have no issues using the resource. Two expressed concerns over technical issues, such as freezing and audio quality. One expressed concern over language barriers. Four expressed concerns regarding the lack of empathy or possible loss of personal touch. One expressed concern over the ability of the avatar to answer the questions required. In total, 20%‐25% expressed some form of apprehension. The prevalence of triggering the wrong conversational stream was not formally measured in the study; however, users did not remark on it when providing open-ended feedback on usability.

*Question: “Any other comments?”*

Two responses:

*good to use at home*

*Needle testing could have been shown twice*

### Missing Data

Those with complete data had higher levels of education, female sex, were less trusting of (*P*=.04) and less satisfied (*P*=.03) with their health care professional than those lost to follow-up (Multimedia Appendix 3). However, the response rates of those in “digital clinician” and the “control” groups at follow-up were similar, as were measures of knowledge, self-efficacy, and usability.

## Discussion

### Principal Findings

Those who received education from the digital clinician were significantly more knowledgeable about their medication and its administration. They tended to have less stress and anxiety associated with using their injections, though this was not significant. Paradoxically, they had less confidence administering injections. They also trusted that their education provider was significantly less than those in the control group and were significantly less satisfied with their consultation. This suggests that from a health care psychology standpoint, participants’ injection-related confidence was more related to who they received their information from, rather than how well informed they were.

While being less satisfied than those in the control group, the intervention group still reported very high levels of trust and satisfaction at levels, with median levels of 7/7 in both measures. Participants were extremely positive about the intervention, with more than 80% of participants expressing that they would use the resource in their own time. If not used clinically, distribution of a “digital clinician” for home use could also add value. The study did not recruit adequate numbers to test noninferiority at the predetermined level of self-efficacy due to global semaglutide shortages hindering patient access. However, there were significant differences in a range of secondary outcomes.

Human clinician-patient interactions are not scripted. They are shaped by human factors, including rapport and variability. Consultations with the “digital clinician” are more uniform and consistent. This may explain why a human was more successful at ensuring trust and satisfaction, while the “digital clinician” was more effective at ensuring information was provided, tested, and retained.

Certain studies have compared physicians versus chatbots, in educational settings, by assessing accuracy. No study has been uncovered showing that patients themselves were better educated by a chatbot or an LLM than by a specialist clinician. It is one thing to assess what information the chatbot is providing; it is another thing to assess if a patient is receiving, retaining, and trusting that information. Moving forward, only the true benefit of “digital clinicians” can be judged when research examines the risks and benefits of integration on a systemic level.

Standards for new technologies should be established to enable regulation, safety, and trust. Bespoke task-specific conversation streams such as this, which mimic large language models, may be easier to control and regulate while taking advantage of growing trust and recognition in the public domain.

Automation may incorporate tradeoffs in patient satisfaction and health care system trust, but may offer benefits in comprehensiveness, efficacy, and uniformity. Tradeoffs could potentially be minimized through research, human oversight, and clear physician accountability. The opportunity cost of using these technologies or not should be considered in terms of productivity, finance, access, and workforce and patient health.

### Limitations

Limitations of the study include the loss of participants to follow-up questioning due to a global shortage of semaglutide, possibly contributing to some of the nonsignificant results in the study. There were significant missing data at the 2-week follow-up, which were omitted from analysis. Table S1 in Multimedia Appendix 4 shows the different profile of responders at the 2-week follow-up. Responders tended to have stronger views and were not a reliable representation of the initial cohort, making the conclusions regarding self-efficacy less applicable to the general population.

Nonvalidated measures of knowledge and adapted measures of usability, trust, and consultation satisfaction were used. While the trial design focuses on intergroup analyses, nonvalidated measures preclude direct comparison of the study cohort with the study population, as existing data for the latter does not exist using the same parameters. As a result, nonvalidated and adapted measures may not correlate well with other health outcomes such as compliance or the incidence of side effects as established measures.

This feasibility trial, while not measuring traditional health outcomes, would have been strengthened by prospective trial registration, a prerequisite for all interventional AI trials from January 01, 2025 [38]. Trial registration was initially omitted from the design process. Contributing factors included the novelty of the intervention and the relative sparsity of an established industry proforma at the time. During the trial and data analysis period, further recognition of AI use as a significant health care intervention led to the emergence of industry directives, prompting reflection and retrospective trial registration. While not included in this study, further longitudinal research could focus on clinical outcomes such as adherence to therapy or weight loss over longer periods (eg, 1 year).

### Conclusion

There is a worsening shortage of health care workers globally. Systems are not adapting to keep up with demand in areas such as bariatric medicine. “Digital clinicians,”chatbots integrated with digital avatar and emotional intelligence technology, may be part of scalable AI solutions.

“Digital clinicians” may ensure higher levels of information retention among patients compared with humans. However, users have significantly lower levels of trust and satisfaction in their information provider. Despite not being as satisfied, users are still overwhelmingly positive about their consultation, with over 80% saying they would use the resource in their own time.

Offering a degree of automation feasibly allows existing health care workers to focus on more demanding tasks, reduce stress, and improve health care system performance. It may offer more patients and systems access to medication and resources, provided they have the necessary internet connection and operability.

Safety should be established through regulation and research to improve trust and satisfaction. Ethical principles such as human oversight and accountability should build on this. Rigorous policy analysis should be performed to assess the potential tradeoffs in using these technologies as AI becomes more widespread across resource-rich and resource-poor contexts.

## Supplementary material

10.2196/63503 Multimedia Appendix 1 Video of digital clinician in use.

10.2196/63503 Multimedia Appendix 2 Outcome measures.

10.2196/63503 Multimedia Appendix 3 Trial protocol.

10.2196/63503 Multimedia Appendix 4 Self-efficacy scores across control and digital clinician groups.

10.2196/63503 Checklist 1 CONSORT-eHEALTH checklist (V 1.6.1).

## Abbreviations

AI: artificial intelligence
CONSORT-AI: Consolidated Standards of Reporting Trials- Artificial Intelligence
SIAQ: Self-Injection Assessment Questionnaire
SPIRIT-AI: S
SPIRIT-AI: Standard Protocol Items: Recommendations for Interventional Trials involving Artificial Intelligence
TUQ: Technology Usability Questionnaire
WHO: World Health Organization
