# Supplementary material for: “Digital Clinicians” Performing Obesity Medication Self-Injection Education: Feasibility Randomized Controlled Trial
Source: JMIR Diabetes. 2025 Jul 30;10:e63503. doi: 10.2196/63503 (PMC12309861; doi:10.2196/63503)
Supplement: Multimedia Appendix 2 [file diabetes-v10-e63503-s002.docx]

**Supplementary Appendix of Outcome Measure Tools**

**Semaglutide Injection Tutorial Assessment: Baseline Characteristics, Knowledge, Self-Efficacy, Trust and Usability**

| **Demographics** |  | | | |
| --- | --- | --- | --- | --- |
| Age |  | | | |
| Gender |  | | | |
| Education Level | None | Primary | Post-Primary | Third Level |
| Ethnicity |  | | | |

**Pre- Tutorial Questionnaire. (1)**

1. In general, how afraid are you of needles?

| Not at all | A little | Moderately | Very | Extremely |
| --- | --- | --- | --- | --- |

1. In general, how afraid are you of having an injection?

| Not at all | A little | Moderately | Very | Extremely |
| --- | --- | --- | --- | --- |

1. How anxious do you feel about giving **yourself** an injection?

| Not at all | A little | Moderately | Very | Extremely |
| --- | --- | --- | --- | --- |

1. How confident are you about giving yourself an injection in **the right way**

| Not at all | A little | Moderately | Very | Extremely |
| --- | --- | --- | --- | --- |

1. How confident are you about giving yourself an injection in a **clean and sterile way**?

| Not at all | A little | Moderately | Very | Extremely |
| --- | --- | --- | --- | --- |

1. How confident are you about giving yourself an injection **safely**?

| Not at all | A little | Moderately | Very | Extremely |
| --- | --- | --- | --- | --- |

7. Overall, how satisfied are you with your current way of taking your medication?

| Very dissatisfied | Dissatisfied | Neither dissatisfied  nor satisfied | Satisfied | Very satisfied |
| --- | --- | --- | --- | --- |

**2. Knowledge Assessment**

Knowledge

Q1.  Which answer is **wrong:** Your new medication

1. Is sometimes called Ozempic
2. Is sometimes called semaglutide
3. Is sometimes called liraglutide
4. Works on GLP-1 receptors

Q2. Your new medication primarily works by?

1. Reducing appetite and causing you to eat less
2. Reducing the intake of fat in the gut
3. Shrinking the stomach to make you feel full
4. Giving you more energy to burn fat

Q3. Where is **not** a recommended injection site?

1. The front of the thigh
2. Directly below the bellybutton
3. The arm
4. Below the bellybutton, to the side

Q4. How often do you fill your pen with medication?

1. Every day
2. Once a month
3. After each use
4. Never

**- POST Tutorial -**

**1. Knowledge Assessment**

**Circle the answer you think is correct**

Knowledge

Q1.  Which answer is **wrong:** Your new medication

1. Is sometimes called Ozempic
2. Is sometimes called semaglutide
3. Is sometimes called liraglutide
4. Works on GLP-1 receptors

Q2. Your new medication primarily works by?

1. Reducing appetite and causing you to eat less
2. Reducing the intake of fat in the gut
3. Shrinking the stomach to make you feel full
4. Giving you more energy to burn fat

Q3. Where is **not** a recommended injection site?

1. The front of the thigh
2. Directly below the bellybutton
3. The arm
4. Below the bellybutton, to the side

Q4. How often do you fill your pen with medication?

1. Every day
2. Once a month
3. After each use
4. Never

Q5. How often do you inject your new medication?

1. Once a day
2. Once every three days
3. Once a week
4. Once every five days

Q6. What dose are you starting on?

1. 0.1mg
2. 0.25mg
3. 0.5mg
4. 1mg

Q7. How often do you change the needle?

1. After each use
2. After you finish using a pen
3. When you increase or lower your dose
4. Only when you think the needle may be dirty

Q8 How should the medication look through the pen window?

1. Red
2. Clear
3. Cloudy
4. Yellow

Q9. What strategy do we recommend to help with side effects?

1. Drink milk
2. Eat slowly
3. Eat more
4. Avoid eating

Q10. What do you do when you miss a dose?

1. Take a double dose
2. Take it anytime before your next dose
3. Take it within five days of missing the dose
4. Only take it the next day or forget about it.

Q11. How often should you have a bowel motion?

1. At least once a week
2. At least three times a week
3. Every day
4. It doesn’t matter

Q12. Where should you store your medication?

1. In the fridge and once open, use within 12 weeks
2. In the fridge and once open, use within 6 weeks
3. In a cool dry, place and once open, use within 12 weeks
4. In a cool, dry, place and once open, use within 6 weeks

**3. Trust- Distrust Measure (2)**

**Please consider the interaction between yourself and the digital nurse. Use an arrow  to indicate your agreement with the following statements:** please place an arrow at any point along the scale below to indicate your agreement).


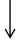
**FOR EXAMPLE:**


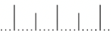


I am suspicious of the digital nurse’s intent or actions

**Not at All**
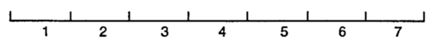
**Extremely**

I have confidence in the digital nurse

**Not at All**
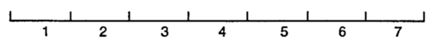
**Extremely**

The digital nurse provided accurate information

**Not at All**
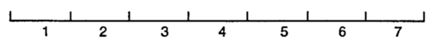
**Extremely**

The digital nurse has integrity

**Not at All**
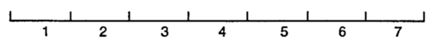
**Extremely**

The digital nurse is trustworthy

**Not at All**
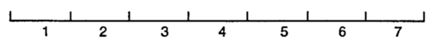
**Extremely**

**4. Satisfaction with Healthcare Professional (3)**

|  | **Strongly**  **Agree** | **Agree** | **Slightly**  **Agree** | **Neutral** | **Slightly Disagree** | **Disagree** | **Strongly Disagree** |
| --- | --- | --- | --- | --- | --- | --- | --- |
| The Digital nurse accurately explained to You, what the education session  involves |  |  |  |  |  |  |  |
| I would like The Digital nurse to be  available at my medical clinic |  |  |  |  |  |  |  |
| The Digital nurse explained things in a  way that was easy to understand |  |  |  |  |  |  |  |
| I am confident of The Digital nurse’s knowledge and skills |  |  |  |  |  |  |  |
| The Digital nurse listened to You |  |  |  |  |  |  |  |
| The Digital nurse cares about You as a person |  |  |  |  |  |  |  |
| The Digital nurse encouraged You to  talk about her health issues |  |  |  |  |  |  |  |
| The Digital nurse spent enough time  with You |  |  |  |  |  |  |  |

**5. Digital Avatar Usability (4)**

| Rate the technology in the following areas from 1 to 5, 5 being excellent and 1 being very poor. | **1** | **2** | | **3** | **4** | **5** |
| --- | --- | --- | --- | --- | --- | --- |
| **Interface Quality** (Audio, visual quality) |  |  | |  |  |  |
| **Interaction Quality** (Quality of conversation, body language, responsiveness, general interaction) |  |  | |  |  |  |
| **How easy it was to use** |  |  | |  |  |  |
| **Future Use-** Please answer the following questions | | | | | | |
| What do you think about using avatars with automated conversation in a healthcare environment? |  | | | | | |
| What concerns would you have about using avatars with automated conversation like this in the future? |  | | | | | |
| Would you use this resource in your own time? Tick the corresponding box | Yes | | No | Maybe | | |
| Any other comments? |  | | | | | |

**2 WEEKS POST TUTORIAL  (1)**

**Feelings about injections**

In general, how afraid are you of needles?

| Not at all | A little | Moderately | Very | Extremely |
| --- | --- | --- | --- | --- |

In general, how afraid are you of having an injection?

| Not at all | A little | Moderately | Very | Extremely |
| --- | --- | --- | --- | --- |

How anxious do you feel about giving **yourself** an injection?

| Not at all | A little | Moderately | Very | Extremely |
| --- | --- | --- | --- | --- |

**Self-image**

How embarrassed would you feel if someone saw you with the self-injection device?

| Not at all | A little | Moderately | Very | Extremely |
| --- | --- | --- | --- | --- |

**Self-confidence**

How confident are you about giving yourself an injection in **the right way**?

| Not at all | A little | Moderately | Very | Extremely |
| --- | --- | --- | --- | --- |

How confident are you about giving yourself an injection in a **clean and sterile way**?

| Not at all | A little | Moderately | Very | Extremely |
| --- | --- | --- | --- | --- |

How confident are you about giving yourself an injection **safely**?

| Not at all | A little | Moderately | Very | Extremely |
| --- | --- | --- | --- | --- |

**Pain and skin reactions during or after the injection**

The following questions ask about **pain and skin reactions** you may have experienced during or after the injection.

Please answer each question below by checking the box that best represents your opinion (Check only one box per question).

| During and/or after the injection, how bothered were you by: | Not at all | A little | Moderately | Very | Extremely |
| --- | --- | --- | --- | --- | --- |
| 1. **pain**? |  |  |  |  |  |
| 1. **burning sensation**? |  |  |  |  |  |
| 1. **cold sensation**? |  |  |  |  |  |

| During and/or after the injection, how bothered were you by: | Not at all | A little | Moderately | Very | Extremely |
| --- | --- | --- | --- | --- | --- |
| 1. **itching** at the   injection site? |  |  |  |  |  |
| 1. **redness** at the injection site? |  |  |  |  |  |
| 1. **swelling** at the injection site? |  |  |  |  |  |
| 1. **bruising** at the injection site? |  |  |  |  |  |
| 1. **hardening** at the injection site? |  |  |  |  |  |

**Ease of Use of the self-injection device**

The following questions ask about the **ease of use** of the self-injection device.

Please answer each question below by checking the box that best represents your opinion (Check only one box per question).

| How difficult or easy was it to: | Very difficult | Difficult | Somewhat difficult | Somewhat easy | Easy | Very easy |
| --- | --- | --- | --- | --- | --- | --- |
| remove the cap? |  |  |  |  |  |  |
| depress the plunger or button on the device? |  |  |  |  |  |  |
| administer the injection without any help? |  |  |  |  |  |  |
| use the self-injection device? |  |  |  |  |  |  |

How does the device fit in your hand?

| Very uncomfortably | Uncomfortably | Somewhat  uncomfortably | Somewhat  comfortably | Comfortably | Very comfortably |
| --- | --- | --- | --- | --- | --- |

**Satisfaction with self-injection**

The following questions ask about your **satisfaction** with self-injection.

Please answer each question below by checking the box that best represents your opinion (Check only one box per question).

How easy was it to give yourself an injection?

| Not at all | A little | Moderately | Very | Extremely |
| --- | --- | --- | --- | --- |

How satisfied are you with **how often** you give yourself an injection?

| Very dissatisfied | Dissatisfied | Neither dissatisfied  nor satisfied | Satisfied | Very satisfied |
| --- | --- | --- | --- | --- |

How satisfied are you with the **time it takes to inject** the medication?

| Very dissatisfied | Dissatisfied | Neither dissatisfied  nor satisfied | Satisfied | Very satisfied |
| --- | --- | --- | --- | --- |

Overall, how satisfied are you with your current way of taking your medication (selfinjection)?

| Very dissatisfied | Dissatisfied | Neither dissatisfied  nor satisfied | Satisfied | Very satisfied |
| --- | --- | --- | --- | --- |

Overall, how convenient is the self-injection device?

| Very  inconvenient | Inconvenient | Neither inconvenient  nor convenient | Convenient | Very convenient |
| --- | --- | --- | --- | --- |

After this study, would you choose to continue self-injecting your medication?

| Definitely not | Probably not | I don’t know | Yes, probably | Yes, definitely |
| --- | --- | --- | --- | --- |

After this study, how confident would you be to give yourself injections at home?

| Not at all | A little | Moderately | Very | Extremely |
| --- | --- | --- | --- | --- |

**Thank you for completing this questionnaire**

References

1. Pompilus F, Ciesluk A, Strzok S, Ciaravino V, Harris K, Szegvari B, et al. Development and psychometric evaluation of the assessment of self-injection questionnaire: an adaptation of the self-injection assessment questionnaire. Health Qual Life Outcomes. 2020;18(1):355.
2. Jiun-Yin Jian, Ann M. Bisantz & Colin G. Drury (2000) Foundations for an Empirically Determined Scale of Trust in Automated Systems, International Journal of Cognitive Ergonomics, 4:1, 53-71, DOI: 10.1207/S15327566IJCE0401_04.
3. Hojat M, Louis DZ, Maxwell K, Markham FW, Wender RC, Gonnella JS. A brief instrument to measure patients' overall satisfaction with primary care physicians. Fam Med. 2011;43(6):412-7.
4. Hajesmaeel-Gohari S, Bahaadinbeigy K. The most used questionnaires for evaluating telemedicine services. BMC Med Inform Decis Mak. 2021;21(1):36.
