# Supplementary material for: “Digital Clinicians” Performing Obesity Medication Self-Injection Education: Feasibility Randomized Controlled Trial
Source: JMIR Diabetes. 2025 Jul 30;10:e63503. doi: 10.2196/63503 (PMC12309861; doi:10.2196/63503)
Supplement: Multimedia Appendix 3 [file diabetes-v10-e63503-s003.pdf]

Study Protocol: As guided by Consort-AI(1) and Spirit-AI Extensions(2)

Title: Obesity Medication Self-Injection Education Using "Digital Clinicians": A Feasibility Randomised Controlled Trial

Principal Invesitgator

Title: Prof. Name: Derek O'Keeffe

Qualifications :

MB BCh BAO GDip (Clin Ed) MD FRCPI

MRCP(UK) (Endocrinology & Diabetes)

B Eng M Eng PhD MIEI

Position : Consultant Physician, Professor of Medical Device Technology.

Dept : Endocrinology.

Organisation : University Hospital Galway, National University of Ireland, Galway.

Address : NUI Galway, Ireland

Tel : +353-871493278 E-mail: derek.okeeffe@nuigalway.ie

What is the anticipated start date of this study?

1st of May 2023

What is the anticipated duration of this study?

3 Months

Brief information on the study background.

In most clinical check-ups and follow-ups, the clinician spends a significant amount of time educating the patients, helping them understand medication, its impact, and the measures to control/prevent the disease. In the field of bariatric medicine, all patients at many centres, are required to receive education and assessment from a clinician specialist on medication administration before discharge. Introducing a "digital clinician"- an AI (Artificial Intelligence) powered avatar- to educate patients will enable Clinicians to concentrate on other areas, solve complex problems related to obesity and ease the workload. A global supply shortage of healthcare workers, is currently exacerbated in certain areas such as bariatric medicine, where novel injectable solutions are not available to swathes of the world's population.

Our proposed idea is to educate patients about beginning their injectable therapy and managing side-effects etc., with the use of a mobile, scalable digital solution. This may

increase the efficiency and efficacy of the system on and individual and systemic level. As well as saving considerable time for Clinicians and patients, clinicians may be able to focus energy on alternative tasks, working “higher up” their license.

An educational session with the “digital clinician “consist of resourceful interactive lessons, photos and videos about various aspects of the drug, injections, risks, expectations and administration. A “digital clinician” will also provide interactive questions and replays of certain videos, if the patient wishes.

A “digital clinician” will be first created for the purpose of this study. It is a virtual avatar representation of a clinician, integrated with language and emotion procession technologies to mimic human interaction. The conversation streams used by the “digital clinician” will be generated for the purpose of this research study only, and focus only on the assigned task, and be clinically approved by the research team before being integrated with the virtual avatar.

This study will randomly allocate education sessions from a “digital clinician” with human oversight and education sessions from a Clinician alone to real patients starting semaglutide pharmacotherapy for the treatment of overweight and obesity for the first time. The study design includes comparing performance metrics for “digital clinicians” Vs Clinician. We want to identify and evaluate different parameters such as knowledge levels, self-efficacy, trust levels and consultation satisfaction levels pre- and post-education session across both education providers- real and AI.

This study aims to analyse feedback from users and broaden its use in other healthcare settings. Like any new introduction into a healthcare setting, technology should be thoroughly and methodically assessed to improve on future iterations.

List the study aims and objectives.

1. To compare patient self-efficacy of participants using the Self Injection Assement Questionnaire (SIAQ) scores. The SIAQ is a validated questionnaire that records a composite of data. The score includes confidence and anxiety levels, comfort, satisfaction with administration, patient assessment of their safety self-administering injections before and after injection administration. It also includes scoring related to the incidence of side-effects after administering injections.

2. Secondary aims are to measure Trust, Consultation satisfaction, knowledge levels pre and post tutorial and technology usability using a range oo validated and non-validated measures. These will be compared across control and intervention groups

This study aims to:

- Study the Feasibility of a digital clinician educating patients about self-administration of semaglutide once-weekly injections for weight loss.
- Study and better understand patients' and healthcare professionals' opinions on digital clinicians and software platforms that are used in health care education.
- Consider potential trade-offs between uniformity, knowledge transfer, trust and consultation satisfaction when using a non-human clinician.

List the study endpoints / measurable outcomes (if applicable).

- Overall SIAQ Post Injection Score- The SIAQ uses scales of 1-4 and 1-5 to assess domains of data from less desirable to more desirable. These scales will be converted to a 10 point scale for equivalency.
- The change from baseline post injection score compared to the pre injection score will also be measured and compared between the control and intervention group.
- Technology Acceptance will be measured using the Technology Usability Questionnaire
- Trust in healthcare provider levels- Jian et al. Trust-Distrust Scale
- Consultation satisfaction- Consultation Satisfaction in Physician Scale
- Knowledge levels pre- and post education session
- All endpoints will be measured across both groups, apart from the technology usability questionnaire which will only be administered to the intervention group.

Provide information on the study design.

The study will be a Non-blinded Single-Centre Randomised Controlled Non-Inferiority Trial of digital clinician delivered education in bariatric patients starting semaglutide injections at the outpatient bariatric medicine clinic in University Hospital Galway.

The participants are the patients in the bariatric clinic starting semaglutide, and health care professionals (HCP).

Stratified Randomisaton will occur based on patients age, BMI and baseline Pre Injection score, and pre-tutorial knowledge scores.

The study will strive to recruit 28 patients' to recruit each arm of the study, 56 patients in total.

Patients will be verbally informed of the research study. Those who decline to formally participate will also be offered the chance to interact with the digital clinician.

Written informed consent will be obtained from those who wish to participate.

The participant will then A) receive the intervention with supervision from a medical student followed by meeting the clinician, or B) the control, education from the clinician only.

Follow up will occur via phone call with HCP at 6-weeks to discuss any patient issues and the email issuance of the Post Injection SIAQ to be completed online and anonymously.

At any point during the study, any participant can choose to withdraw.

After collection of all the study data, outcome variables will be measured.

Provide information on the study methodology.

Once a decision has been reached that a patient will begin daily self-administered semaglutide injections, they return to the the waiting area before being called for education on their new drug. These patients will be identified to a medical student or participating HCP. After discussion and permission of the appropriate governing bodies/individuals of the healthcare setting (i.e., Consultant in charge, Nurse manager etc), the patients; and staff would be invited to participate in the research study. Willing participants will be handed the Patient Information Leaflet and consent form

The patient will be given 30 minutes to decide on their participation. Written informed consent will be obtained. If they are willing to take part, they will fill in the Pre -Injection section of the SIAQ.

Randomisation using minimisation will be used with a pre-defined formula to minimise differences across age, BMI, and pre tutorial knowledge and self-efficacy levels. Excel will be used to calculate a score based on these four factors and particpiants will be deterministically placed into the group which minimises the differences between these variables between groups at the point of entry into the study.

Patients assigned to the control group will then interact on a 1:1 basis with the digital clinicain. A medical student will be present who is familiar with the technology for questions. The tutorial will last approximately 15 minutes. The digital clinician will play videos and ask the patient questions.

The videos will be recorded in advance of the study by HCP's in the bariatric clinic, UHG. The videos will include all the information they deem important, information they include in every consultation for beginning self-administered semaglutide injections. The HCPs and patients will be involved in designing the interactions to include details they deem important and necessary.

After completing the education session with the digital clinician, patients will then meet a HCP who will assess their knowledge and answer any questions they might have before being deemed safe for discharge.

Patients randomised to the control group will only meet the HCP and receive a normal consultation, typically lasting 15 minutes. Patients will be assured to contact the clinic or their G.P if they encounter any issues or if they have any problems.

After discharge, patients will be contacted in 6 weeks via telephone and email. The HCP will assess satisfaction and answer queries over the phone while they will receive a link to an anonymised post-injection section of the SIAQ via email. Survey monkey will be used to obtain this data.

Mean, standard deviations and 95% confidence intervals of the primary outcomes will be calculated and non-inferiority will be tested. The Technology Usability questionnaire will be used to assess the acceptance of technology in this role and compared to data from previous studies.

At any point during the study, any participant can choose to withdraw from the study.

Provide information on the statistical approach to be used in the analysis of your results (if appropriate) / source of any statistical advice.

The SIAQ is validated and there are several papers reporting summary statistics. This paper (Table 4) finds an SD of 1.5 (Keininger et al, 2011). Categorisation of the highest scorers and lowest scorers show a mean difference on the 10-point scale of between one and 2. Consultants in the bariatric clinic and the Primary and Co-Invesigators of the Research Project decided that a margin of two-thirds of the significant difference, d=1, would demonstrate non-inferiority.

Non-inferiority was chosen as oppose to superiority. The research question involves improving the administration of care, and whether there is space for Digital clinician and chatbots/avatars in healthcare education. Non-inferiority would justify cost-benefit analysis, analysis of wait times, workload and job satisfaction etc. to name a few. Technology will also improve as times goes on and although we do not expect Digital Clinician to outperform a HCP, there are still possibly benefits in showing he is as good as a HCP.

T-tests will be used to assess superiority across the secondary outcome variables. In the case of non-normal data, a non-parametric t-test will be used such as the Mann-Whitney U test.

Please justify the proposed sample size and provide details of its calculation (including minimum clinically important difference).

Using the website, https://www.sealedenvelope.com/power/continuous-noninferior/, a calculator recommended from a paper- Sample sizes for clinical trials with Normal data (Julius et al, 2004).

The following calculation was made.

Significance level (alpha)- 5%

Power (1-beta)- 80%

Standard deviation of outcome-1.5, Non-inferiority limit **d=1,**

Calculation formula

$n = f(\alpha, \beta) \times 2 \times \sigma^2 / d^2$

where σ is the standard deviation, and

$f(\alpha, \beta) = [\Phi^{-1}(\alpha) + \Phi^{-1}(\beta)]^2$

Φ^{-1} is the cumulative distribution function of a standardised normal deviate.

Sample size required per group- 28

Total sample size required- 56

<u>How many</u> research participants are to be recruited <u>in total</u>?

56

<u>How many</u> research participants are to be recruited <u>in each study group</u> (where applicable)? Please complete the following table (where applicable).

**Name of Study Group:**	**Name of Study Group:**
Intervention	Control
**Number of Participants in this Study Group:**	**Number of Participants in this Study Group:**
28	28

Please provide details on the method of randomisation (where applicable).

Minimisation by randomisation will be used. Allocations were independently assessed using the below formula which was absolute. Participants were assigned one by one in order of study enrolment to the group which minimised differences across four pre-determined baseline variables. Allocations were concealed until after baseline data was collected. Assignment was unchangeable at this point. Microsoft Excel was used to allocate participants one-by-one using the below formula by an assessor at a separate location.

$$((AGE-20)/4)+((BMI-20)/2)+((\text{Knowledge Score}/4)*15)+((\text{Self-Efficacy}/35)*15)$$

Knowledge and self-efficacy were scored out of 4 and 35 respectively. Assuming an age range of 20-80 years, and BMI range of 25 to 60, scores were based on scales of 0-15 for age, knowledge and self-efficacy, and 2.5-20 for BMI. BMI had a greater impact on allocation due to the increased anxiety associated with healthcare consultations and higher BMI.

Allocation is deterministic and unknown until the point of allocation. An assessor at a different location to the trial participants will receive the relevant information and utilise the formula on a computer software before assessing allocation. The formula result will be absoloute, and hence, concealed.

How many research participants are to be recruited at each study site (where applicable)? Please complete the following table.

Site:	Number of Research Participants at this site:
Galway	56

PARTICIPANTS – SELECTION AND RECRUITMENT

How will the participants in the study be selected?

Once a decision has been reached that a patient will begin daily self-administered semaglutide injections, they return to the the waiting area before being called for education on their new drug. After discussion and permission of the appropriate governing bodies/individuals of the healthcare setting (i.e., Consultant in charge, Nurse manager etc), the patients; and staff would be invited to participate in the research study. Willing participants will be handed the Patient Information Leaflet and consent form.

All patients starting semaglutide self-administered injections during the duration of the study will be invited to participate until 56 patients are recruited.

How will the participants in the study be recruited?

These patients will be identified to a medical student or participating HCP. These patients will be approached and asked to privately asked to participate in the research study. The patient will be offered the Patient Information Leaflet and consent Form. Patients will be given 30 minutes to decide whether or not they would like to participate in the research project. Clinical staff will also be approached via normal methods of departmental communications (i.e., team meetings, email updates, via clinical managers etc.) and invited to participate in the research study.

What are the inclusion criteria for research participants? (Please justify, where necessary)

To be included in the research study, patients must be starting semaglutide self-injection administration, for the first time and awaiting education. They must have capacity to give consent to being involved in the study, and judged to be capable to provide self-administration of injections by the human clinicians in the clinic- this will be determined in advance of being referred for education.

What are the exclusion criteria for research participants? (Please justify, where necessary)

- A Critically ill patients, who do not have capacity to consent or participate in the surveys due to their medical status (will be determined by the clinical care team)
- While patients diagnosed with dementia or cognitive impairment will not be automatically excluded, participants must be able to give fully informed consent and can participate in completing the survey.

Data Inputs and Outputs

Data input by the users into the tutorial is available for recording on the IBM Watson software, but this will be turned off and will be inaccessible to anyone after the participant's use. Outputs from the AI will not be recorded either. This is to minimise the exposure of privacy of the participants in the trial.

The outputs of the "digital clinician" did not alter the course of clinical decision making. The human clinician oversaw the encounter by meeting the patient after the session and assessing their knowledge/safety briefly before discharge.

Code

The code for the "digital clinician" will be made available after the study and will accompany the submission of manuscript to various journals in the supplementary material.

Performance Errors

Performance errors will be identified through supervision of the AI education session and rectification will be reported to the engineering members of the research team who will work to rectify them if the team feels it is ethically necessary throughout the study. Any changes will be recorded in the limitations of data thereafter.

Participants will be advised after knowledge assessments are made of any errors that have been made. Note will be made, and all errors will be included in publication of the study.

Results

On completion of the study, 43 participants were recruited, 27 to the intervention group and 16 to the control group. Patients in the "digital clinician" group were significantly more knowledgeable post-consultation ($p<0.001$) and had better feelings about their injections ($p=.079$). Patients in the control group were more satisfied with their consultation ($p<0.001$) and had more trust in their education provider ($p<0.001$). There was no significant difference in reported levels of self-efficacy. 81% of participants said they would use the resource in their own time.

Further details can be found upon publication or through contacting the research team.

1. Liu X, Cruz Rivera S, Moher D, Calvert MJ, Denniston AK, Spirit AI, et al. Reporting guidelines for clinical trial reports for interventions involving artificial intelligence: the CONSORT-AI extension. Nat Med. 2020;26(9):1364-74.
2. Rivera SC, Liu X, Chan AW, Denniston AK, Calvert MJ, Spirit AI, et al. Guidelines for clinical trial protocols for interventions involving artificial intelligence: the SPIRIT-AI Extension. BMJ. 2020;370:m3210.
