# Supplementary material for: “Digital Clinicians” Performing Obesity Medication Self-Injection Education: Feasibility Randomized Controlled Trial
Source: JMIR Diabetes. 2025 Jul 30;10:e63503. doi: 10.2196/63503 (PMC12309861; doi:10.2196/63503)
Supplement: Multimedia Appendix 4 [file diabetes-v10-e63503-s004.pdf]

Table 1: Missing Data

Baseline Characteristics	Incomplete Cases (n=28)	Complete cases (n=15)	
	Mean(SD)		p-value
Age	46.9(11.8)	49.9(13.6)	.489
BMI	44.2(9.1)	41.8(5.5)	.360
Gender	n(%)		
Male	7(25.0)	2(12.5)	
Female	21(75.0)	14(87.5)	
Ethnicity			
Irish	17 (63.0)	13 (81.3)	
Other	3 (11.1)	1 (6.3)	
Missing	7 (25.9)	2 (22.2)	
Education level			
None	1 (3.6)	0 (0.0)	
Primary	2 (7.1)	0 (0.0)	
Secondary	14 (50.0)	5 (33.3)	
Third Level	21 (39.3)	10 (66.6)	
	Median (Interquartile Range)		
Pre-Tutorial Knowledge	2.0 (1.0-2.0)	2.0 (1.0-3.0)	.348
Pre-Tutorial Self Efficacy	31.0 (27.0-32.5)	32.0 (28.0-33.5)	.521
**Group Allocation**	n(%)		
Digital Clinician	17 (60.7)	10 (66.7)	
Control	11 (39.3)	5 (33.3)	
**Outcomes**			
Self-Efficacy	NA	NA	NA
Knowledge	Raw Score (/12)		
Overall Knowledge	10.0(8.0-11.0)	10.0 (9.0-11.0)	0.494
Trust-Distrust Scale	Raw Score (/7)		
Overall Trust	7.0 (6.0-7.0)	6.0 (6.0-7.0)	.044
Consultation Satisfaction	Raw Score (/7)(Mean Rank)		
Overall Satisfaction	7.0 (6.0-7.0) (202)	7.0 (6.0-7.0)(179)	.025
	Incomplete (n=17)	Complete (n=10)	
Usability	Raw Score (/5)		
Overall	5.0 (4.0-5.0)	4.0 (4.0-5.0)	0.340
Would you use/ this Resource in your own time?	n(%)		
Yes	9 (90)	13 (76)	
No	1 (10)	1 (6.0)	
Maybe	0 (0)	3 (18)	

Mann-Whitney U test used to calculate p-value
