# Supplementary material for: “Digital Clinicians” Performing Obesity Medication Self-Injection Education: Feasibility Randomized Controlled Trial
Source: JMIR Diabetes. 2025 Jul 30;10:e63503. doi: 10.2196/63503 (PMC12309861; doi:10.2196/63503)
Supplement: Checklist 1 [file diabetes-v10-e63503-s005.pdf]

CONSORT-EHEALTH (V 1.6.1) - Submission/Publication Form

The CONSORT-EHEALTH checklist is intended for authors of randomized trials evaluating web-based and Internet-based applications/interventions, including mobile interventions, electronic games (incl multiplayer games), social media, certain telehealth applications, and other interactive and/or networked electronic applications. Some of the items (e.g. all subitems under item 5 - description of the intervention) may also be applicable for other study designs.

The goal of the CONSORT EHEALTH checklist and guideline is to be
a) a guide for reporting for authors of RCTs,
b) to form a basis for appraisal of an ehealth trial (in terms of validity)

CONSORT-EHEALTH items/subitems are MANDATORY reporting items for studies published in the Journal of Medical Internet Research and other journals / scientific societies endorsing the checklist.

Items numbered 1., 2., 3., 4a., 4b etc are original CONSORT or CONSORT-NPT (non-pharmacologic treatment) items.
Items with Roman numerals (i., ii, iii, iv etc.) are CONSORT-EHEALTH extensions/clarifications.

As the CONSORT-EHEALTH checklist is still considered in a formative stage, we would ask that you also RATE ON A SCALE OF 1-5 how important/useful you feel each item is FOR THE PURPOSE OF THE CHECKLIST and reporting guideline (optional).

Mandatory reporting items are marked with a red *.
In the textboxes, either copy & paste the relevant sections from your manuscript into this form - please include any quotes from your manuscript in QUOTATION MARKS,
or answer directly by providing additional information not in the manuscript, or elaborating on why the item was not relevant for this study.

YOUR ANSWERS WILL BE PUBLISHED AS A SUPPLEMENTARY FILE TO YOUR PUBLICATION IN JMIR AND ARE CONSIDERED PART OF YOUR PUBLICATION (IF ACCEPTED).
Please fill in these questions diligently. Information will not be copyedited, so please use proper spelling and grammar, use correct capitalization, and avoid abbreviations.

DO NOT FORGET TO SAVE AS PDF _AND_ CLICK THE SUBMIT BUTTON SO YOUR ANSWERS ARE IN OUR DATABASE !!!

Citation Suggestion (if you append the pdf as Appendix we suggest to cite this paper in the caption):
Eysenbach G, CONSORT-EHEALTH Group

CONSORT-EHEALTH: Improving and Standardizing Evaluation Reports of Web-based and Mobile Health Interventions
J Med Internet Res 2011;13(4):e126
URL: http://www.jmir.org/2011/4/e126/
doi: 10.2196/jmir.1923
PMID: 22209829

Sign in to Google to save your progress. Learn more

* Indicates required question

Your name *

First Last

Seán Coleman

Primary Affiliation (short), City, Country *

University of Toronto, Toronto, Canada

University Hospital Galway, Galway, Ireland

Your e-mail address *

abc@gmail.com

seancoleman@live.ie

Title of your manuscript *

Provide the (draft) title of your manuscript.

“Digital Clinicians” performing OBESITY Medication Self-Injection Education: A Feasibility Randomised Controlled Trial

Name of your App/Software/Intervention *

If there is a short and a long/alternate name, write the short name first and add the long name in brackets.

Digital Clinician (Using SoulMachines)

Evaluated Version (if any)

e.g. "V1", "Release 2017-03-01", "Version 2.0.27913"

Version 1

Language(s) *

What language is the intervention/app in? If multiple languages are available, separate by comma (e.g. "English, French")

English

URL of your Intervention Website or App

e.g. a direct link to the mobile app on app in appstore (itunes, Google Play), or URL of the website. If the intervention is a DVD or hardware, you can also link to an Amazon page.

Your answer

URL of an image/screenshot (optional)

https://www.soulmachines.com/

Accessibility *

Can an enduser access the intervention presently?

- ( ) access is free and open
- (•) access only for special usergroups, not open
- ( ) access is open to everyone, but requires payment/subscription/in-app purchases
- ( ) app/intervention no longer accessible
- ( ) Other:

Primary Medical Indication/Disease/Condition *

e.g. "Stress", "Diabetes", or define the target group in brackets after the condition, e.g. "Autism (Parents of children with)", "Alzheimers (Informal Caregivers of)"

Overweight/Obesity

Primary Outcomes measured in trial *

comma-separated list of primary outcomes reported in the trial

Self-efficacy

Secondary/other outcomes

Are there any other outcomes the intervention is expected to affect?

Knowledge of medication, Trust, Usability, Consultation Satisfaction

Recommended "Dose" *

What do the instructions for users say on how often the app should be used?

- ( ) Approximately Daily
- ( ) Approximately Weekly
- ( ) Approximately Monthly
- ( ) Approximately Yearly
- (•) "as needed"
- ( ) Other:

Approx. Percentage of Users (starters) still using the app as recommended after 3 months *

- ( ) unknown / not evaluated
- ( ) 0-10%
- ( ) 11-20%
- ( ) 21-30%
- ( ) 31-40%
- ( ) 41-50%
- ( ) 51-60%
- ( ) 61-70%
- ( ) 71%-80%
- ( ) 81-90%
- ( ) 91-100%
- (•) Other: One off intervention

Overall, was the app/intervention effective? *

- ○ yes: all primary outcomes were significantly better in intervention group vs control
- ◉ partly: SOME primary outcomes were significantly better in intervention group vs control
- ○ no statistically significant difference between control and intervention
- ○ potentially harmful: control was significantly better than intervention in one or more outcomes
- ○ inconclusive: more research is needed
- ○ Other:

Article Preparation Status/Stage *

At which stage in your article preparation are you currently (at the time you fill in this form)

- ○ not submitted yet - in early draft status
- ○ not submitted yet - in late draft status, just before submission
- ○ submitted to a journal but not reviewed yet
- ◉ submitted to a journal and after receiving initial reviewer comments
- ○ submitted to a journal and accepted, but not published yet
- ○ published
- ○ Other:

Journal *

If you already know where you will submit this paper (or if it is already submitted), please provide the journal name (if it is not JMIR, provide the journal name under "other")

- ○ not submitted yet / unclear where I will submit this
- ○ Journal of Medical Internet Research (JMIR)
- ○ JMIR mHealth and UHealth
- ○ JMIR Serious Games
- ○ JMIR Mental Health
- ○ JMIR Public Health
- ○ JMIR Formative Research
- ◉ Other JMIR sister journal
- ○ Other:

Is this a full powered effectiveness trial or a pilot/feasibility trial? *

- ◉ Pilot/feasibility
- ○ Fully powered

Manuscript tracking number *

If this is a JMIR submission, please provide the manuscript tracking number under "other" (The ms tracking number can be found in the submission acknowledgement email, or when you login as author in JMIR. If the paper is already published in JMIR, then the ms tracking number is the four-digit number at the end of the DOI, to be found at the bottom of each published article in JMIR)

- ○ no ms number (yet) / not (yet) submitted to / published in JMIR
- ◉ Other: #63503

TITLE AND ABSTRACT

1a) TITLE: Identification as a randomized trial in the title

1a) Does your paper address CONSORT item 1a? *

I.e does the title contain the phrase "Randomized Controlled Trial"? (if not, explain the reason under "other")

- (selected) yes
- Other:

1a-i) Identify the mode of delivery in the title

Identify the mode of delivery. Preferably use “web-based” and/or “mobile” and/or “electronic game” in the title. Avoid ambiguous terms like “online”, “virtual”, “interactive”. Use “Internet-based” only if Intervention includes non-web-based Internet components (e.g. email), use “computer-based” or “electronic” only if offline products are used. Use “virtual” only in the context of “virtual reality” (3-D worlds). Use “online” only in the context of “online support groups”. Complement or substitute product names with broader terms for the class of products (such as “mobile” or “smart phone” instead of “iphone”), especially if the application runs on different platforms.

	1	2	3	4	5	
subitem not at all important	○	○	○	●	○	essential

Clear selection

Does your paper address subitem 1a-i? *

Copy and paste relevant sections from manuscript title (include quotes in quotation marks "like this" to indicate direct quotes from your manuscript), or elaborate on this item by providing additional information not in the ms, or briefly explain why the item is not applicable/relevant for your study

"digital clinicians" is a term coined during the research project for virtual humans performing clinical tasks and is included in the title. It is clarified in the abstract and manuscript.

1a-ii) Non-web-based components or important co-interventions in title

Mention non-web-based components or important co-interventions in title, if any (e.g., “with telephone support”).

	1	2	3	4	5	
subitem not at all important	○	○	◉	○	○	essential

Clear selection

Does your paper address subitem 1a-ii?

Copy and paste relevant sections from manuscript title (include quotes in quotation marks "like this" to indicate direct quotes from your manuscript), or elaborate on this item by providing additional information not in the ms, or briefly explain why the item is not applicable/relevant for your study

N/A

Your answer must have a minimum of 25 characters.

1a-iii) Primary condition or target group in the title

Mention primary condition or target group in the title, if any (e.g., “for children with Type I Diabetes”) Example: A Web-based and Mobile Intervention with Telephone Support for Children with Type I Diabetes: Randomized Controlled Trial

	1	2	3	4	5	
subitem not at all important	○	○	○	○	○	essential

Does your paper address subitem 1a-iii? *

Copy and paste relevant sections from manuscript title (include quotes in quotation marks "like this" to indicate direct quotes from your manuscript), or elaborate on this item by providing additional information not in the ms, or briefly explain why the item is not applicable/relevant for your study

Yes, it is used for "obesity self-injection medication"

1b) ABSTRACT: Structured summary of trial design, methods, results, and conclusions

NPT extension: Description of experimental treatment, comparator, care providers, centers, and blinding status.

1b-i) Key features/functionalities/components of the intervention and comparator in the METHODS section of the ABSTRACT

Mention key features/functionalities/components of the intervention and comparator in the abstract. If possible, also mention theories and principles used for designing the site. Keep in mind the needs of systematic reviewers and indexers by including important synonyms. (Note: Only report in the abstract what the main paper is reporting. If this information is missing from the main body of text, consider adding it)

	1	2	3	4	5	
subitem not at all important	○	○	○	●	○	essential

Clear selection

Does your paper address subitem 1b-i? *

Copy and paste relevant sections from the manuscript abstract (include quotes in quotation marks "like this" to indicate direct quotes from your manuscript), or elaborate on this item by providing additional information not in the ms, or briefly explain why the item is not applicable/relevant for your study

"This study investigates the use of a chatbot integrated with a digital avatar to create a “digital clinician”. This was used to provide mandatory patient education for those beginning semaglutide once-weekly self-administered injections for the treatment of overweight and obesity at a national centre.
Methods: A “Digital Clinician” with facial and vocal recognition technology was generated with a bespoke 10-to-15-minute clinician-validated tutorial. " It was compared to a clinical nurse specialist performing the same task, as detailed in the manuscript and abstract.

1b-ii) Level of human involvement in the METHODS section of the ABSTRACT

Clarify the level of human involvement in the abstract, e.g., use phrases like “fully automated” vs. “therapist/nurse/care provider/physician-assisted” (mention number and expertise of providers involved, if any). (Note: Only report in the abstract what the main paper is reporting. If this information is missing from the main body of text, consider adding it)

	1	2	3	4	5	
subitem not at all important	○	○	○	◉	○	essential

Clear selection

Does your paper address subitem 1b-ii?

Copy and paste relevant sections from the manuscript abstract (include quotes in quotation marks "like this" to indicate direct quotes from your manuscript), or elaborate on this item by providing additional information not in the ms, or briefly explain why the item is not applicable/relevant for your study

"between those using the AI-powered clinician avatar and those receiving conventional semaglutide education from nursing staff. Attitudes were recorded immediately after the intervention and again at two-weeks after the education session."

1b-iii) Open vs. closed, web-based (self-assessment) vs. face-to-face assessments in the METHODS section of the ABSTRACT

Mention how participants were recruited (online vs. offline), e.g., from an open access website or from a clinic or a closed online user group (closed usergroup trial), and clarify if this was a purely web-based trial, or there were face-to-face components (as part of the intervention or for assessment). Clearly say if outcomes were self-assessed through questionnaires (as common in web-based trials). Note: In traditional offline trials, an open trial (open-label trial) is a type of clinical trial in which both the researchers and participants know which treatment is being administered. To avoid confusion, use “blinded” or “unblinded” to indicated the level of blinding instead of “open”, as “open” in web-based trials usually refers to “open access” (i.e. participants can self-enrol). (Note: Only report in the abstract what the main paper is reporting. If this information is missing from the main body of text, consider adding it)

	1	2	3	4	5	
subitem not at all important	○	○	◉	○	○	essential

Clear selection

Does your paper address subitem 1b-iii?

Copy and paste relevant sections from the manuscript abstract (include quotes in quotation marks "like this" to indicate direct quotes from your manuscript), or elaborate on this item by providing additional information not in the ms, or briefly explain why the item is not applicable/relevant for your study

Assessed in a face to face outpatient clinic directly after a doctors consultation where once weekly injections are prescribed and education is received.
"A feasibility randomised controlled non-inferiority trial compared knowledge test scores, self-efficacy, consultation satisfaction, and trust levels between those using the AI-powered clinician avatar on-site and those receiving conventional semaglutide education from nursing staff. Attitudes were recorded immediately after the intervention and again at two-weeks after the education session."

1b-iv) RESULTS section in abstract must contain use data

Report number of participants enrolled/assessed in each group, the use/uptake of the intervention (e.g., attrition/adherence metrics, use over time, number of logins etc.), in addition to primary/secondary outcomes. (Note: Only report in the abstract what the main paper is reporting. If this information is missing from the main body of text, consider adding it)

	1	2	3	4	5	
subitem not at all important	○	○	○	●	○	essential

Clear selection

Does your paper address subitem 1b-iv?

Copy and paste relevant sections from the manuscript abstract (include quotes in quotation marks "like this" to indicate direct quotes from your manuscript), or elaborate on this item by providing additional information not in the ms, or briefly explain why the item is not applicable/relevant for your study

": 43 participants were recruited, 27 to the intervention group and 16 to the control group. Patients in the “digital clinician” group were significantly more knowledgeable post-consultation (IQR 10.0-11.0 vs 7.0-9.3, (p<0.001). and had better feelings about their injections (p=.079). Patients in the control group were more satisfied with their consultation (IQR 6.0-7.0 vs 7.0-7.0, p<0.001) and had more trust in their education provider (IQR 4.8-7.0 vs 7.0-7.0, p<0.001). There was no significant difference in reported levels of self-efficacy (p=.57). 81% (22 of 27)of participants in the intervention group said they would use the resource in their own time."

1b-v) CONCLUSIONS/DISCUSSION in abstract for negative trials

Conclusions/Discussions in abstract for negative trials: Discuss the primary outcome - if the trial is negative (primary outcome not changed), and the intervention was not used, discuss whether negative results are attributable to lack of uptake and discuss reasons. (Note: Only report in the abstract what the main paper is reporting. If this information is missing from the main body of text, consider adding it)

	1	2	3	4	5	
subitem not at all important	○	○	◉	○	○	essential

Clear selection

Does your paper address subitem 1b-v?

Copy and paste relevant sections from the manuscript abstract (include quotes in quotation marks "like this" to indicate direct quotes from your manuscript), or elaborate on this item by providing additional information not in the ms, or briefly explain why the item is not applicable/relevant for your study

Not addressed drectly in the abstract but in the body of the manuscript "The study did not recruit adequate numbers to test non-inferiority at the pre-determined level of self-efficacy due to global semaglutide shortages hindering patient access. However, there were significant differences in a range of secondary outcomes."

INTRODUCTION

2a) In INTRODUCTION: Scientific background and explanation of rationale

2a-i) Problem and the type of system/solution

Describe the problem and the type of system/solution that is object of the study: intended as stand-alone intervention vs. incorporated in broader health care program? Intended for a particular patient population? Goals of the intervention, e.g., being more cost-effective to other interventions, replace or complement other solutions? (Note: Details about the intervention are provided in “Methods” under 5)

	1	2	3	4	5	
subitem not at all important	○	○	○	◉	○	essential

Clear selection

Does your paper address subitem 2a-i? *

Copy and paste relevant sections from the manuscript (include quotes in quotation marks "like this" to indicate direct quotes from your manuscript), or elaborate on this item by providing additional information not in the ms, or briefly explain why the item is not applicable/relevant for your study

"Obesity, an "abnormal or excessive accumulation of fat that poses a health risk", [(1]) is a primary contributor to global health challenges ([2]). Reports suggest its involvement in up to 80% of cases of type 2 diabetes and 43% of cardiovascular incidents ([3]) while significantly contributing to depression and anxiety ([4]).
Recent breakthroughs in bariatric medicine have elevated the role of injectable therapy, namely Glucagon Like Peptide-1 (GLP-1) agonist agents such as semaglutide. New injectable agents are showing weight loss surpassing 20% and 25% ([5]). With over half of adults in the WHO European Region ([2]) potentially eligible, utility is hindered by scalability of clinical services and supply. Generative AI has been identified to the potential to offset the clinical and administrative demands associated with the management of patients on these medication types [6]
"Healthcare systems are underresourced and understaffed, particularly in rural areas ([87]). This impacts productivity, sustainability ([98]) and the health of both patient and healthcare professional ([109]). Healthcare worker shortages, propelled by aging populations ([1110]) and the Covid-19 pandemic ([1211]) are exacerbated in areas such as the Global South, increasing healthcare inequities ([132]). Tasks that healthcare workers perform are often repetitive and administrative. Redistribution of such tasks has been shown to potentially improve health outcomes such as blood pressure, HbA1c, and mental health ([143]).
"

2a-ii) Scientific background, rationale: What is known about the (type of) system

Scientific background, rationale: What is known about the (type of) system that is the object of the study (be sure to discuss the use of similar systems for other conditions/diagnoses, if appropiate), motivation for the study, i.e. what are the reasons for and what is the context for this specific study, from which stakeholder viewpoint is the study performed, potential impact of findings [2]. Briefly justify the choice of the comparator.

	1	2	3	4	5	
subitem not at all important	○	○	◉	○	○	essential

Clear selection

Does your paper address subitem 2a-ii? *

Copy and paste relevant sections from the manuscript (include quotes in quotation marks "like this" to indicate direct quotes from your manuscript), or elaborate on this item by providing additional information not in the ms, or briefly explain why the item is not applicable/relevant for your study

"Artificial Intelligence (AI) Chatbots have the potential to be used for a variety of medical tasks including note taking ([154]) and personalised medicine ([165]). Anonymised AI chatbots have been judged to provide better, more concise, empathetic answers to general health queries than verified physicians ([176]). Chat Generative Pre-Trained Transformer(GPT)-3 is known to be accurate with common chief complaints ([187]) and GPT-4 recently outscored 99.98% of simulated human readers when diagnosing complex clinical cases ([198]). Automated personalised messaging systems are being researched to enhance behavioural change in a hope to enhance health outcomes [20]"

2b) In INTRODUCTION: Specific objectives or hypotheses

Does your paper address CONSORT subitem 2b? *

Copy and paste relevant sections from the manuscript (include quotes in quotation marks "like this" to indicate direct quotes from your manuscript), or elaborate on this item by providing additional information not in the ms, or briefly explain why the item is not applicable/relevant for your study

"The aim of thisis study is to investigates the use of a medically-approved task-specific “digital clinician” to provide patient education in a clinical environment, comparing it with a human counterpart." Hypothesis is that it will perform non-inferiorly to a human counterpart."

METHODS

3a) Description of trial design (such as parallel, factorial) including allocation ratio

Does your paper address CONSORT subitem 3a? *

Copy and paste relevant sections from the manuscript (include quotes in quotation marks "like this" to indicate direct quotes from your manuscript), or elaborate on this item by providing additional information not in the ms, or briefly explain why the item is not applicable/relevant for your study

Your answer

3b) Important changes to methods after trial commencement (such as eligibility criteria), with reasons

Does your paper address CONSORT subitem 3b? *

Copy and paste relevant sections from the manuscript (include quotes in quotation marks "like this" to indicate direct quotes from your manuscript), or elaborate on this item by providing additional information not in the ms, or briefly explain why the item is not applicable/relevant for your study

"A randomised controlled non-inferiority trial of "digital clinician"-led patient education was designed with ethical approval from Galway University Hospitals (GUH), Clinical Research Ethics Committee (Ref: CA 2920). The trial and trial protocol were guided by SPIRIT-AI[28] and CONSORT-AI[29] extensions.
Further information on the study protocol can be found in the supplementary appendix.
All patients at GUH must attend an education session with a Clinical Nurse Specialist before starting semaglutide injectable therapy. This session covers basic semaglutide pharmacotherapy and the safe self-administration of injections using a semaglutide pen.
During the study period, allocated, eligible participants received their mandatory education from a "digital clinician" with human oversight. The control group received current standard-of-care, human, nurse-led education.
Once a clinical decision was made that treatment of overweight or obesity with semaglutide injections would be commenced, eligibility was assessed. Overweight and obesity were classified as a BMI greater than 25 kg/m2.

"

3b-i) Bug fixes, Downtimes, Content Changes

Bug fixes, Downtimes, Content Changes: ehealth systems are often dynamic systems. A description of changes to methods therefore also includes important changes made on the intervention or comparator during the trial (e.g., major bug fixes or changes in the functionality or content) (5-iii) and other "unexpected events" that may have influenced study design such as staff changes, system failures/downtimes, etc. [2].

	1	2	3	4	5	
subitem not at all important	○	○	●	○	○	essential

Clear selection

Does your paper address subitem 3b-i?

Copy and paste relevant sections from the manuscript (include quotes in quotation marks "like this" to indicate direct quotes from your manuscript), or elaborate on this item by providing additional information not in the ms, or briefly explain why the item is not applicable/relevant for your study

Users were supervised to aid with any issues that would arise and this is addressed in the manuscript. No issues arose.

4a) Eligibility criteria for participants

Does your paper address CONSORT subitem 4a? *

Copy and paste relevant sections from the manuscript (include quotes in quotation marks "like this" to indicate direct quotes from your manuscript), or elaborate on this item by providing additional information not in the ms, or briefly explain why the item is not applicable/relevant for your study

Eligibility criteria included being over 18 years of age, without an intellectual or physical disability which would interfere with a participant's ability to self-administer semaglutide injections. All eligible patients were invited to participate in the study. Given the nature of the intervention ("digital clinician"), blinding was not feasible.

4a-i) Computer / Internet literacy

Computer / Internet literacy is often an implicit "de facto" eligibility criterion - this should be explicitly clarified.

	1	2	3	4	5	
subitem not at all important	○	○	◉	○	○	essential

Clear selection

Does your paper address subitem 4a-i?

Copy and paste relevant sections from the manuscript (include quotes in quotation marks "like this" to indicate direct quotes from your manuscript), or elaborate on this item by providing additional information not in the ms, or briefly explain why the item is not applicable/relevant for your study

Not part of the eligibility criteria.

4a-ii) Open vs. closed, web-based vs. face-to-face assessments:

Open vs. closed, web-based vs. face-to-face assessments: Mention how participants were recruited (online vs. offline), e.g., from an open access website or from a clinic, and clarify if this was a purely web-based trial, or there were face-to-face components (as part of the intervention or for assessment), i.e., to what degree got the study team to know the participant. In online-only trials, clarify if participants were quasi-anonymous and whether having multiple identities was possible or whether technical or logistical measures (e.g., cookies, email confirmation, phone calls) were used to detect/prevent these.

	1	2	3	4	5	
subitem not at all important	○	○	●	○	○	essential

Clear selection

Does your paper address subitem 4a-ii? *

Copy and paste relevant sections from the manuscript (include quotes in quotation marks "like this" to indicate direct quotes from your manuscript), or elaborate on this item by providing additional information not in the ms, or briefly explain why the item is not applicable/relevant for your study

All face to face as mentioned above, with supervised use of the software.

4a-iii) Information giving during recruitment

Information given during recruitment. Specify how participants were briefed for recruitment and in the informed consent procedures (e.g., publish the informed consent documentation as appendix, see also item X26), as this information may have an effect on user self-selection, user expectation and may also bias results.

	1	2	3	4	5	
subitem not at all important	○	○	●	○	○	essential

Clear selection

Does your paper address subitem 4a-iii?

Copy and paste relevant sections from the manuscript (include quotes in quotation marks "like this" to indicate direct quotes from your manuscript), or elaborate on this item by providing additional information not in the ms, or briefly explain why the item is not applicable/relevant for your study

Included in application for ethical approval which is referenced in the protocol which is uploaded as supplementary appendix. Few details but only reference in the main manuscript.

4b) Settings and locations where the data were collected

Does your paper address CONSORT subitem 4b? *

Copy and paste relevant sections from the manuscript (include quotes in quotation marks "like this" to indicate direct quotes from your manuscript), or elaborate on this item by providing additional information not in the ms, or briefly explain why the item is not applicable/relevant for your study

Yes at an obesity and weight management outpatient physician clinic.

4b-i) Report if outcomes were (self-)assessed through online questionnaires

Clearly report if outcomes were (self-)assessed through online questionnaires (as common in web-based trials) or otherwise.

	1	2	3	4	5	
subitem not at all important	○	○	◉	○	○	essential

Clear selection

Does your paper address subitem 4b-i? *

Copy and paste relevant sections from the manuscript (include quotes in quotation marks "like this" to indicate direct quotes from your manuscript), or elaborate on this item by providing additional information not in the ms, or briefly explain why the item is not applicable/relevant for your study

N/A- no online surveys were used.

4b-ii) Report how institutional affiliations are displayed

Report how institutional affiliations are displayed to potential participants [on ehealth media], as affiliations with prestigious hospitals or universities may affect volunteer rates, use, and reactions with regards to an intervention.(Not a required item – describe only if this may bias results)

	1	2	3	4	5	
subitem not at all important	○	○	◉	○	○	essential

Clear selection

Does your paper address subitem 4b-ii?

Copy and paste relevant sections from the manuscript (include quotes in quotation marks "like this" to indicate direct quotes from your manuscript), or elaborate on this item by providing additional information not in the ms, or briefly explain why the item is not applicable/relevant for your study

"Logos of affiliated educational and healthcare institutions were included on the User Interface Screen to increase trust. Cinematic Screening offered different angles of the avatars face to make for a more interesting experience."

5) The interventions for each group with sufficient details to allow replication, including how and when they were actually administered

5-i) Mention names, credential, affiliations of the developers, sponsors, and owners

Mention names, credential, affiliations of the developers, sponsors, and owners [6] (if authors/evaluators are owners or developer of the software, this needs to be declared in a “Conflict of interest” section or mentioned elsewhere in the manuscript).

	1	2	3	4	5	
subitem not at all important	○	○	○	●	○	essential

Clear selection

Does your paper address subitem 5-i?

Copy and paste relevant sections from the manuscript (include quotes in quotation marks "like this" to indicate direct quotes from your manuscript), or elaborate on this item by providing additional information not in the ms, or briefly explain why the item is not applicable/relevant for your study

No developers, sponsors or owners were involved in the running of the trial. A 3rd party app was used. Academic affiliations are included as usual and were declared during informed consent.

5-ii) Describe the history/development process

Describe the history/development process of the application and previous formative evaluations (e.g., focus groups, usability testing), as these will have an impact on adoption/use rates and help with interpreting results.

	1	2	3	4	5	
subitem not at all important	○	○	○	○	◉	essential

Clear selection

Does your paper address subitem 5-ii?

Copy and paste relevant sections from the manuscript (include quotes in quotation marks "like this" to indicate direct quotes from your manuscript), or elaborate on this item by providing additional information not in the ms, or briefly explain why the item is not applicable/relevant for your study

"The software IBM Watson Assistant was used to generate conversation streams. Microsoft ClipChamp Video Editor was used to edit videos, then accessed online via source code written on IBM Watson. The IBM Watson conversation code file was integrated with an animated avatar on the SoulMachines Creator Website. The “digital clinician” uses Natural Language Processing and an evolving bank of AI-driven animation responses that monitor vocal tone and facial expression to regulate behaviour. The “digital clinician” has a programmed personality, name, voice and identity and is being used in a role traditionally considered to require human intelligence."
"over 100 iterations of the digital clinician" were generated to maximise cohesion in the development process.

5-iii) Revisions and updating

Revisions and updating. Clearly mention the date and/or version number of the application/intervention (and comparator, if applicable) evaluated, or describe whether the intervention underwent major changes during the evaluation process, or whether the development and/or content was "frozen" during the trial. Describe dynamic components such as news feeds or changing content which may have an impact on the replicability of the intervention (for unexpected events see item 3b).

	1	2	3	4	5	
subitem not at all important	○	○	○	○	◉	essential

Clear selection

Does your paper address subitem 5-iii?

Copy and paste relevant sections from the manuscript (include quotes in quotation marks "like this" to indicate direct quotes from your manuscript), or elaborate on this item by providing additional information not in the ms, or briefly explain why the item is not applicable/relevant for your study

As above..........................

5-iv) Quality assurance methods

Provide information on quality assurance methods to ensure accuracy and quality of information provided [1], if applicable.

	1	2	3	4	5	
subitem not at all important	○	○	○	○	◉	essential

Clear selection

Does your paper address subitem 5-iv?

Copy and paste relevant sections from the manuscript (include quotes in quotation marks "like this" to indicate direct quotes from your manuscript), or elaborate on this item by providing additional information not in the ms, or briefly explain why the item is not applicable/relevant for your study

"All possible conversation streams had been verified against official pharmaceutical literature."

5-v) Ensure replicability by publishing the source code, and/or providing screenshots/screen-capture video, and/or providing flowcharts of the algorithms used

Ensure replicability by publishing the source code, and/or providing screenshots/screen-capture video, and/or providing flowcharts of the algorithms used. Replicability (i.e., other researchers should in principle be able to replicate the study) is a hallmark of scientific reporting.

	1	2	3	4	5	
subitem not at all important	○	○	○	●	○	essential

Clear selection

Does your paper address subitem 5-v?

Copy and paste relevant sections from the manuscript (include quotes in quotation marks "like this" to indicate direct quotes from your manuscript), or elaborate on this item by providing additional information not in the ms, or briefly explain why the item is not applicable/relevant for your study

code published as supplementary appendix

5-vi) Digital preservation

Digital preservation: Provide the URL of the application, but as the intervention is likely to change or disappear over the course of the years; also make sure the intervention is archived (Internet Archive, webcitation.org, and/or publishing the source code or screenshots/videos alongside the article). As pages behind login screens cannot be archived, consider creating demo pages which are accessible without login.

	1	2	3	4	5	
subitem not at all important	○	○	◉	○	○	essential

Clear selection

Does your paper address subitem 5-vi?

Copy and paste relevant sections from the manuscript (include quotes in quotation marks "like this" to indicate direct quotes from your manuscript), or elaborate on this item by providing additional information not in the ms, or briefly explain why the item is not applicable/relevant for your study

Not a publicly available intervention

5-vii) Access

Access: Describe how participants accessed the application, in what setting/context, if they had to pay (or were paid) or not, whether they had to be a member of specific group. If known, describe how participants obtained "access to the platform and Internet" [1]. To ensure access for editors/reviewers/readers, consider to provide a "backdoor" login account or demo mode for reviewers/readers to explore the application (also important for archiving purposes, see vi).

	1	2	3	4	5	
subitem not at all important	○	○	◉	○	○	essential

Clear selection

Does your paper address subitem 5-vii? *

Copy and paste relevant sections from the manuscript (include quotes in quotation marks "like this" to indicate direct quotes from your manuscript), or elaborate on this item by providing additional information not in the ms, or briefly explain why the item is not applicable/relevant for your study

"All patients at GUH must attend an education session with a Clinical Nurse Specialist before starting semaglutide injectable therapy. This session covers basic semaglutide pharmacotherapy and the safe self-administration of injections using a semaglutide pen. During the study period, allocated, eligible participants received their mandatory education from a “digital clinician” with human oversight. The control group received current standard-of-care, human, nurse-led education.
Once a clinical decision was made that treatment of overweight or obesity with semaglutide injections would be commenced, eligibility was assessed. Overweight and obesity were classified as a BMI greater than 25 kg/m2."

5-viii) Mode of delivery, features/functionalities/components of the intervention and comparator, and the theoretical framework

Describe mode of delivery, features/functionalities/components of the intervention and comparator, and the theoretical framework [6] used to design them (instructional strategy [1], behaviour change techniques, persuasive features, etc., see e.g., [7, 8] for terminology). This includes an in-depth description of the content (including where it is coming from and who developed it) [1],” whether [and how] it is tailored to individual circumstances and allows users to track their progress and receive feedback” [6]. This also includes a description of communication delivery channels and – if computer-mediated communication is a component – whether communication was synchronous or asynchronous [6]. It also includes information on presentation strategies [1], including page design principles, average amount of text on pages, presence of hyperlinks to other resources, etc. [1].

	1	2	3	4	5	
subitem not at all important	○	○	◉	○	○	essential

Clear selection

Does your paper address subitem 5-viii? *

Copy and paste relevant sections from the manuscript (include quotes in quotation marks "like this" to indicate direct quotes from your manuscript), or elaborate on this item by providing additional information not in the ms, or briefly explain why the item is not applicable/relevant for your study

Yes.
"To inform design, clinicians were observed educating patients. Their mannerisms, behaviour and conversations were recorded. Transcripts of educational sessions given by the local Obesity Nurse Specialist were generated. A publicly available Patient Information Leaflet and an online video from the manufacturer on medication administration were collated and transcribed.
A 10-to-15 minute educational script was generated with multiple conversation streams. Information on medication name, dosages, mechanism of action, pen preparation, administration, side effects and storage were included. Interactive questions were used to maximise engagement and knowledge retention. The “digital clinician” was programmed to give a limited number of pre-determined responses only.
Multiple conversation streams were generated to reflect the most effective course for an educational session. Akin to natural clinical education, the “digital clinician” led the primary section of the tutorial, offering information and asking the patient questions throughout to test retention and assess understanding. The questions asked tested information retention and assessed understanding. The question types used by the “digital clinician” varied. Some were open, while others were multiple choice, true or false, yes or no, or numerical. After the clinician-led portion of the tutorial had ended, patients were invited to ask questions of their own accord. Users could type or voice any question they wished. The option to see and hear the answers to some Frequently Asked Questions was also offered by way of onscreen prompts. Responses were selected by the “digital clinician” based on the presence of trigger words provided by the user during the interaction. Trigger words were pre-selected and responses were pre-written by the research team using information from the named resources
Unclear or ambiguous responses were identified by the digital clinician as so, this was communicated to the user who was then kindly asked to repeat themselves. When assessing understanding, ifIf not understanding the userstood twice-in-a-row, the “digital clinician” would simply offer the correct answer and continue. This prevented the possibility of an infinite loop. When offered the chance to ask general queries, the user may query infinitely if they wish, although a comprehensive list of Frequently Asked Question prompts on screen, and an onscreen button to end the tutorial, were in place to reduce this need.
A careful selection of trigger words and combinations of IF, AND, OR and NOT statements were used to design the conversational flow. Rigorous internal testing including over 100 iterations of the conversational flow were tested by the research team to ensure a fluent, cohesive, usable digital clinician with little risk of misinterpreting the user’s intent.
The interaction was safety netted somewhat by human oversight and the interaction with the human obesity specialist nurse after the tutorial was complete, offering the chance to patients to clarify any queries that may have not been appropriately addressed.
This “digital clinician” could be accessed with native web browsers on the participants phone, tablet or laptop. In this case, the participants accessed the session under supervision on an iPad device.
Two multi-scene videos recorded by the team and integrated into the tutorial using screen-in-screen projection. The videos offered human views and instructions of what preparing and using the injection pen entailed. The videos, while being demonstrated by members of the research team, were narrated by the “digital clinician”. A set of frequently asked questions was included for optional revision.
The software IBM Watson Assistant was used to generate conversation streams. Microsoft ClipChamp Video Editor was used to edit videos, then accessed online via source code [illegible]

written on IBM Watson. The IBM Watson conversation code file was integrated with an animated avatar on the SoulMachines Creator Website. The “digital clinician” uses Natural Language Processing and an evolving bank of AI-driven animation responses that monitor vocal tone and facial expression to regulate behaviour. The “digital clinician” has a programmed personality, name, voice and identity and is being used in a role traditionally considered to require human intelligence.
Logos of affiliated educational and healthcare institutions were included on the User Interface Screen to increase trust. Cinematic Screening offered different angles of the avatars face to make for a more interesting experience.
Multiple avatars of different genders, race and name were used to promote inclusivity.
All possible conversation streams had been verified against official pharmaceutical literature. The individual scripts were not recorded, but each participant-AI interaction was supervised to monitor for technical issues or hallucinations.
"

5-ix) Describe use parameters

Describe use parameters (e.g., intended “doses” and optimal timing for use). Clarify what instructions or recommendations were given to the user, e.g., regarding timing, frequency, heaviness of use, if any, or was the intervention used ad libitum.

	1	2	3	4	5	
subitem not at all important	○	○	◉	○	○	essential

Clear selection

Does your paper address subitem 5-ix?

Copy and paste relevant sections from the manuscript (include quotes in quotation marks "like this" to indicate direct quotes from your manuscript), or elaborate on this item by providing additional information not in the ms, or briefly explain why the item is not applicable/relevant for your study

As above.................

5-x) Clarify the level of human involvement

Clarify the level of human involvement (care providers or health professionals, also technical assistance) in the e-intervention or as co-intervention (detail number and expertise of professionals involved, if any, as well as “type of assistance offered, the timing and frequency of the support, how it is initiated, and the medium by which the assistance is delivered”. It may be necessary to distinguish between the level of human involvement required for the trial, and the level of human involvement required for a routine application outside of a RCT setting (discuss under item 21 – generalizability).

	1	2	3	4	5	
subitem not at all important	○	○	◉	○	○	essential

Clear selection

Does your paper address subitem 5-x?

Copy and paste relevant sections from the manuscript (include quotes in quotation marks "like this" to indicate direct quotes from your manuscript), or elaborate on this item by providing additional information not in the ms, or briefly explain why the item is not applicable/relevant for your study

As detailed above, humans were in the room to offer help if needed, but at no point were they needed throughout use of the intervention. This is explained in the results and discussion sections.

5-xi) Report any prompts/reminders used

Report any prompts/reminders used: Clarify if there were prompts (letters, emails, phone calls, SMS) to use the application, what triggered them, frequency etc. It may be necessary to distinguish between the level of prompts/reminders required for the trial, and the level of prompts/reminders for a routine application outside of a RCT setting (discuss under item 21 – generalizability).

	1	2	3	4	5	
subitem not at all important	○	◉	○	○	○	essential

Clear selection

Does your paper address subitem 5-xi? *

Copy and paste relevant sections from the manuscript (include quotes in quotation marks "like this" to indicate direct quotes from your manuscript), or elaborate on this item by providing additional information not in the ms, or briefly explain why the item is not applicable/relevant for your study

Not relevant as this was a once off use of an app, under supervision.

5-xii) Describe any co-interventions (incl. training/support)

Describe any co-interventions (incl. training/support): Clearly state any interventions that are provided in addition to the targeted eHealth intervention, as ehealth intervention may not be designed as stand-alone intervention. This includes training sessions and support [1]. It may be necessary to distinguish between the level of training required for the trial, and the level of training for a routine application outside of a RCT setting (discuss under item 21 – generalizability.

	1	2	3	4	5	
subitem not at all important	(•)	( )	( )	( )	( )	essential

Clear selection

Does your paper address subitem 5-xii? *

Copy and paste relevant sections from the manuscript (include quotes in quotation marks "like this" to indicate direct quotes from your manuscript), or elaborate on this item by providing additional information not in the ms, or briefly explain why the item is not applicable/relevant for your study

No training or support was used or needed.

6a) Completely defined pre-specified primary and secondary outcome measures, including how and when they were assessed

Does your paper address CONSORT subitem 6a? *

Copy and paste relevant sections from the manuscript (include quotes in quotation marks "like this" to indicate direct quotes from your manuscript), or elaborate on this item by providing additional information not in the ms, or briefly explain why the item is not applicable/relevant for your study

2.4.1 Self-Efficacy
Self-efficacy was measured using the two-part validated Self Injection Assessment Questionnaire (SIAQ) ([3126]). The pre-injection SIAQ was used as baseline data.
The post-injection SIAQ was scored across five domains two-weeks post-intervention- feelings about injections, self-image, self-confidence, pain and skin reactions, ease of use and satisfaction.
The SIAQ has been shown to be a valid robust tool with sufficient validity, reliability, consistency and sensitivity. Cronbach's alpha and the test-retest coefficient were > 0.70 for all domains. ([3227]).
2.4.2 Knowledge Attainment
Unvalidated knowledge assessments were designed specifically for the purpose of this research.
The pre-educational assessment tool consisted of four questions. The post-educational assessment tool contained the same initial four questions, and a further eight questions. Questions were multiple choice and covered topics such as drug name, drug class, mechanism of action, side effects, injection technique and storage requirements.
2.4.3 Consultation Satisfaction
The validated Patients' Overall Satisfaction with Primary Care Physicians Scale ([3328]) has Criterion-related validity coefficients mostly in the 0.80s and 0.90s when considering empathy, physician recommendation and general satisfaction. Cronbach's coefficient alpha for the patient satisfaction scale is 0.98. The 7-point-likert-scale was used in the study.
2.4.4 Trust
The “Trust between People and Automation Scale” has undergone validation ([3429]) and been in use for over 20 years. It was adapted and integrated with the consultation satisfaction measure for this study.
2.4.5 Technology Usability and Future Use (TUQ)
The validated TUQ ([3530]) was adapted. The questionnaire has high reliability with Cronbach Coefficient Alpha’s over 0.8 across all domains, and high validity expressed through its multiple native questionnaires (3530). A shortened version including questions from four of five domains was used. The domains were “Ease of use and Learnability”, “Interface Quality”, “Interaction Quality”, and “Satisfaction and future use”. The TUQ had previously measured the usability of a Droid Audio-Visual Educator (59).
Open-ended questions were used to record general themes and attitudes towards the “digital clinician”.

6a-i) Online questionnaires: describe if they were validated for online use and apply CHERRIES items to describe how the questionnaires were designed/deployed

If outcomes were obtained through online questionnaires, describe if they were validated for online use and apply CHERRIES items to describe how the questionnaires were designed/deployed [9].

	1	2	3	4	5	
subitem not at all important	◉	○	○	○	○	essential

Clear selection

Does your paper address subitem 6a-i?

Copy and paste relevant sections from manuscript text

Not applicable to this study.

6a-ii) Describe whether and how “use” (including intensity of use/dosage) was defined/measured/monitored

Describe whether and how “use” (including intensity of use/dosage) was defined/measured/monitored (logins, logfile analysis, etc.). Use/adoption metrics are important process outcomes that should be reported in any ehealth trial.

	1	2	3	4	5	
subitem not at all important	◉	○	○	○	○	essential

Clear selection

Does your paper address subitem 6a-ii?

Copy and paste relevant sections from manuscript text

Use was not measured in this trial as the intervention was a one off.

6a-iii) Describe whether, how, and when qualitative feedback from participants was obtained

Describe whether, how, and when qualitative feedback from participants was obtained (e.g., through emails, feedback forms, interviews, focus groups).

	1	2	3	4	5	
subitem not at all important	○	○	○	○	○	essential

Does your paper address subitem 6a-iii?

Copy and paste relevant sections from manuscript text

Feedback forms which were given on site in form of a questionnaire adapted as described above.

6b) Any changes to trial outcomes after the trial commenced, with reasons

Does your paper address CONSORT subitem 6b? *

Copy and paste relevant sections from the manuscript (include quotes in quotation marks "like this" to indicate direct quotes from your manuscript), or elaborate on this item by providing additional information not in the ms, or briefly explain why the item is not applicable/relevant for your study

No....................................

7a) How sample size was determined

NPT: When applicable, details of whether and how the clustering by care provides or centers was addressed

7a-i) Describe whether and how expected attrition was taken into account when calculating the sample size

Describe whether and how expected attrition was taken into account when calculating the sample size.

	1	2	3	4	5	
subitem not at all important	○	○	●	○	○	essential

Clear selection

Does your paper address subitem 7a-i?

Copy and paste relevant sections from manuscript title (include quotes in quotation marks "like this" to indicate direct quotes from your manuscript), or elaborate on this item by providing additional information not in the ms, or briefly explain why the item is not applicable/relevant for your study

Your answer

7b) When applicable, explanation of any interim analyses and stopping guidelines

Does your paper address CONSORT subitem 7b? *

Copy and paste relevant sections from the manuscript (include quotes in quotation marks "like this" to indicate direct quotes from your manuscript), or elaborate on this item by providing additional information not in the ms, or briefly explain why the item is not applicable/relevant for your study

When calculating sample size, a similar study using the validated Self-Injection Assessment Questionnaire (SIAQ) to measure self-efficacy was used (2926). It showed a Standard Deviation of 1.5, on a 10 point-likert scale. A significant difference of two-thirds the standard deviation was proposed to boundary non-inferiority, with 80% power and alpha equals to 0.05, this would require 28 participants to be recruited into each arm of the study.

8a) Method used to generate the random allocation sequence
NPT: When applicable, how care providers were allocated to each trial group

Does your paper address CONSORT subitem 8a? *

Copy and paste relevant sections from the manuscript (include quotes in quotation marks "like this" to indicate direct quotes from your manuscript), or elaborate on this item by providing additional information not in the ms, or briefly explain why the item is not applicable/relevant for your study

"Minimisation is a method employed in clinical trials and experimental research to allocate participants. ([3025])([4])). Participants were allocated in order of enrolment, to the study arm which minimised the difference across four selected numerical baseline variables- Age, BMI, pre-tutorial knowledge of semaglutide score and pre-tutorial injection self-efficacy score. Running averages of the enrolled participants data were recorded cumulatively for each arm New participants were then allocated to the study arm, which minimised the discrepancy of the running averages between groups. Order of enrolment in the study was determined by check-in time at the clinic. Allocation by minimisation is deterministic and hence precludes traditional concealment.
The first participant was assigned at random using a coin toss. The second participant was subsequently assigned to the alternative group.
Allocation was verified using Microsoft Excel by an independent assessor with access to study data in real-time at a separate location via a secure online suppository. The assessor then communicated the allocation to the research team on site."

8b) Type of randomisation; details of any restriction (such as blocking and block size)

Does your paper address CONSORT subitem 8b? *

Copy and paste relevant sections from the manuscript (include quotes in quotation marks "like this" to indicate direct quotes from your manuscript), or elaborate on this item by providing additional information not in the ms, or briefly explain why the item is not applicable/relevant for your study

"Minimisation is a method employed in clinical trials and experimental research to allocate participants.)([4])). Participants were allocated in order of enrolment, to the study arm which minimised the difference across four selected numerical baseline variables- Age, BMI, pre-tutorial knowledge of semaglutide score and pre-tutorial injection self-efficacy score. Running averages of the enrolled participants data were recorded cumulatively for each arm New participants were then allocated to the study arm, which minimised the discrepancy of the running averages between groups. Order of enrolment in the study was determined by check-in time at the clinic. Allocation by minimisation is deterministic and hence precludes traditional concealment.
The first participant was assigned at random using a coin toss. The second participant was subsequently assigned to the alternative group.
Allocation was verified using Microsoft Excel by an independent assessor with access to study data in real-time at a separate location via a secure online suppository. The assessor then communicated the allocation to the research team on site."

9) Mechanism used to implement the random allocation sequence (such as sequentially numbered containers), describing any steps taken to conceal the sequence until interventions were assigned

Does your paper address CONSORT subitem 9? *

Copy and paste relevant sections from the manuscript (include quotes in quotation marks "like this" to indicate direct quotes from your manuscript), or elaborate on this item by providing additional information not in the ms, or briefly explain why the item is not applicable/relevant for your study

"Minimisation is deterministic and hence precludes traditional concealment"

10) Who generated the random allocation sequence, who enrolled participants, and who assigned participants to interventions

Does your paper address CONSORT subitem 10? *

Copy and paste relevant sections from the manuscript (include quotes in quotation marks "like this" to indicate direct quotes from your manuscript), or elaborate on this item by providing additional information not in the ms, or briefly explain why the item is not applicable/relevant for your study

One assessor recorded baseline variables and an independent assessor used microsft excel to assign participants at a different site- as included above.

11a) If done, who was blinded after assignment to interventions (for example, participants, care providers, those assessing outcomes) and how

NPT: Whether or not administering co-interventions were blinded to group assignment

11a-i) Specify who was blinded, and who wasn't

Specify who was blinded, and who wasn't. Usually, in web-based trials it is not possible to blind the participants [1, 3] (this should be clearly acknowledged), but it may be possible to blind outcome assessors, those doing data analysis or those administering co-interventions (if any).

	1	2	3	4	5	
subitem not at all important	(●)	( )	( )	( )	( )	essential

Clear selection

Does your paper address subitem 11a-i? *

Copy and paste relevant sections from the manuscript (include quotes in quotation marks "like this" to indicate direct quotes from your manuscript), or elaborate on this item by providing additional information not in the ms, or briefly explain why the item is not applicable/relevant for your study

Blinding was not feasible given the nature of the intervention. This is stated in the manuscript.

11a-ii) Discuss e.g., whether participants knew which intervention was the “intervention of interest” and which one was the “comparator”

Informed consent procedures (4a-ii) can create biases and certain expectations - discuss e.g., whether participants knew which intervention was the “intervention of interest” and which one was the “comparator”.

	1	2	3	4	5	
subitem not at all important	○	○	○	○	◉	essential

Clear selection

Does your paper address subitem 11a-ii?

Copy and paste relevant sections from the manuscript (include quotes in quotation marks "like this" to indicate direct quotes from your manuscript), or elaborate on this item by providing additional information not in the ms, or briefly explain why the item is not applicable/relevant for your study

This was known to participants.

11b) If relevant, description of the similarity of interventions

(this item is usually not relevant for ehealth trials as it refers to similarity of a placebo or sham intervention to a active medication/intervention)

Does your paper address CONSORT subitem 11b? *

Copy and paste relevant sections from the manuscript (include quotes in quotation marks "like this" to indicate direct quotes from your manuscript), or elaborate on this item by providing additional information not in the ms, or briefly explain why the item is not applicable/relevant for your study

They were not similar, this is not addressed in the manuscript.

12a) Statistical methods used to compare groups for primary and secondary outcomes

NPT: When applicable, details of whether and how the clustering by care providers or centers was addressed

Does your paper address CONSORT subitem 12a? *

Copy and paste relevant sections from the manuscript (include quotes in quotation marks "like this" to indicate direct quotes from your manuscript), or elaborate on this item by providing additional information not in the ms, or briefly explain why the item is not applicable/relevant for your study

"Data was analysed using Jamovi version 2.4.14 and R Studio ([361, 372]). All Likert scale data were appropriately aggregated in treatment and control arms and recalibrated to scales from 1-to-10 for self-efficacy, and 1-to-7 for trust, satisfaction and usability.
The Mann-Whitney U test was used to assess differences in primary and secondary outcome variables. This nonparametric test is robust to the distribution of the outcome data. The Mann Whitney U test compares the ranks of all the data points in two groups. P-values below 0.05 were deemed statistically significant. Conventional Content analysis was used to interpret qualitative data.
Missing outcome data were omitted from analysis. Observed outcome data were analysed as if representative of the entire cohort.
Knowledge, Satisfaction, Trust and Usability were measured immediately post-tutorial. Subsequent loss to follow-up at two-weeks did not affect the handling of this data.
Those lost to follow-up were unavailable for self-efficacy analysis. The profile of those with complete data and those with missing data was explored in Table 2."

12a-i) Imputation techniques to deal with attrition / missing values

Imputation techniques to deal with attrition / missing values: Not all participants will use the intervention/comparator as intended and attrition is typically high in ehealth trials. Specify how participants who did not use the application or dropped out from the trial were treated in the statistical analysis (a complete case analysis is strongly discouraged, and simple imputation techniques such as LOCF may also be problematic [4]).

	1	2	3	4	5	
subitem not at all important	○	○	●	○	○	essential

Clear selection

Does your paper address subitem 12a-i? *

Copy and paste relevant sections from the manuscript (include quotes in quotation marks "like this" to indicate direct quotes from your manuscript), or elaborate on this item by providing additional information not in the ms, or briefly explain why the item is not applicable/relevant for your study

"The profile of those with complete data and those with missing data was explored in Table 2." "Thos with complete data had higher levels of education, female gender, were less trusting of (p=.044) and less satisfied (p=.025) with their healthcare professional than those lost to follow-up- see Supplementary Appendix 3.Table 2. However, the response rates of those in “digital clinician” and the “control” groups at follow-up were similar, as were measures of knowledge, self-efficacy and usability. "

12b) Methods for additional analyses, such as subgroup analyses and adjusted analyses

Does your paper address CONSORT subitem 12b? *

Copy and paste relevant sections from the manuscript (include quotes in quotation marks "like this" to indicate direct quotes from your manuscript), or elaborate on this item by providing additional information not in the ms, or briefly explain why the item is not applicable/relevant for your study

Subgroup and adjusted analyses was not focused on, only inter group analyses.

X26) REB/IRB Approval and Ethical Considerations [recommended as subheading under "Methods"] (not a CONSORT item)

X26-i) Comment on ethics committee approval

	1	2	3	4	5	
subitem not at all important	○	○	○	○	●	essential

Clear selection

Does your paper address subitem X26-i?

Copy and paste relevant sections from the manuscript (include quotes in quotation marks "like this" to indicate direct quotes from your manuscript), or elaborate on this item by providing additional information not in the ms, or briefly explain why the item is not applicable/relevant for your study

"A randomised controlled non-inferiority trial of “digital clinician”-led patient education was designed with ethical approval from Galway University Hospitals (GUH), Clinical Research Ethics Committee (Ref: CA 2920). The trial and trial protocol were guided by SPIRIT-AI[28] and CONSORT-AI[29] extensions."

x26-ii) Outline informed consent procedures

Outline informed consent procedures e.g., if consent was obtained offline or online (how? Checkbox, etc.?), and what information was provided (see 4a-ii). See [6] for some items to be included in informed consent documents.

	1	2	3	4	5	
subitem not at all important	○	○	○	●	○	essential

Clear selection

Does your paper address subitem X26-ii?

Copy and paste relevant sections from the manuscript (include quotes in quotation marks "like this" to indicate direct quotes from your manuscript), or elaborate on this item by providing additional information not in the ms, or briefly explain why the item is not applicable/relevant for your study

The paper does not focus on it, however it is included in the study protocol, in the supplementary appendix.

X26-iii) Safety and security procedures

Safety and security procedures, incl. privacy considerations, and any steps taken to reduce the likelihood or detection of harm (e.g., education and training, availability of a hotline)

	1	2	3	4	5	
subitem not at all important	○	○	○	◉	○	essential

Clear selection

Does your paper address subitem X26-iii?

Copy and paste relevant sections from the manuscript (include quotes in quotation marks "like this" to indicate direct quotes from your manuscript), or elaborate on this item by providing additional information not in the ms, or briefly explain why the item is not applicable/relevant for your study

a one off supervised intervention, on an on-site device.

RESULTS

13a) For each group, the numbers of participants who were randomly assigned, received intended treatment, and were analysed for the primary outcome
NPT: The number of care providers or centers performing the intervention in each group and the number of patients treated by each care provider in each center

Does your paper address CONSORT subitem 13a? *

Copy and paste relevant sections from the manuscript (include quotes in quotation marks "like this" to indicate direct quotes from your manuscript), or elaborate on this item by providing additional information not in the ms, or briefly explain why the item is not applicable/relevant for your study

"During the 7-week study period in the bariatric clinic, 53 patients were prescribed semaglutide self-administered injections for the treatment of overweight or obesity. Of this cohort, 43 agreed to participate in the study. Of those enrolled, 100% (43 of 43) completed the pre-tutorial questionnaire, their assigned education session and their post-tutorial questionnaire. Only 18 participants, 41.9%, completed the two-week follow-up self-efficacy questionnaire."

13b) For each group, losses and exclusions after randomisation, together with reasons

Does your paper address CONSORT subitem 13b? (NOTE: Preferably, this is shown in a CONSORT flow diagram) *

Copy and paste relevant sections from the manuscript (include quotes in quotation marks "like this" to indicate direct quotes from your manuscript), or elaborate on this item by providing additional information not in the ms, or briefly explain why the item is not applicable/relevant for your study

" Only 18 participants, 41.9%, completed the two-week follow-up self-efficacy questionnaire."
"The study did not recruit adequate numbers to test non-inferiority at the pre-determined level of self-efficacy due to global semaglutide shortages hindering patient access. However, there were significant differences in a range of secondary outcomes."

13b-i) Attrition diagram

Strongly recommended: An attrition diagram (e.g., proportion of participants still logging in or using the intervention/comparator in each group plotted over time, similar to a survival curve) or other figures or tables demonstrating usage/dose/engagement.

	1	2	3	4	5	
subitem not at all important	○	◉	○	○	○	essential

Clear selection

Does your paper address subitem 13b-i?

Copy and paste relevant sections from the manuscript or cite the figure number if applicable (include quotes in quotation marks "like this" to indicate direct quotes from your manuscript), or elaborate on this item by providing additional information not in the ms, or briefly explain why the item is not applicable/relevant for your study

N/A........................

14a) Dates defining the periods of recruitment and follow-up

Does your paper address CONSORT subitem 14a? *

Copy and paste relevant sections from the manuscript (include quotes in quotation marks "like this" to indicate direct quotes from your manuscript), or elaborate on this item by providing additional information not in the ms, or briefly explain why the item is not applicable/relevant for your study

Not formally stated in the manuscript but detailed in the study protocol.

14a-i) Indicate if critical “secular events” fell into the study period

Indicate if critical “secular events” fell into the study period, e.g., significant changes in Internet resources available or “changes in computer hardware or Internet delivery resources”

	1	2	3	4	5	
subitem not at all important	○	○	●	○	○	essential

Clear selection

Does your paper address subitem 14a-i?

Copy and paste relevant sections from the manuscript (include quotes in quotation marks "like this" to indicate direct quotes from your manuscript), or elaborate on this item by providing additional information not in the ms, or briefly explain why the item is not applicable/relevant for your study

N/A they did not occur.....

14b) Why the trial ended or was stopped (early)

Does your paper address CONSORT subitem 14b? *

Copy and paste relevant sections from the manuscript (include quotes in quotation marks "like this" to indicate direct quotes from your manuscript), or elaborate on this item by providing additional information not in the ms, or briefly explain why the item is not applicable/relevant for your study

N/a................................

15) A table showing baseline demographic and clinical characteristics for each group

NPT: When applicable, a description of care providers (case volume, qualification, expertise, etc.) and centers (volume) in each group

Does your paper address CONSORT subitem 15? *

Copy and paste relevant sections from the manuscript (include quotes in quotation marks "like this" to indicate direct quotes from your manuscript), or elaborate on this item by providing additional information not in the ms, or briefly explain why the item is not applicable/relevant for your study

A table is included in the manuscript.
Table 1
Baseline Characteristics Total (n=43) Digital Clinician (n=27) Control (n=16)
Mean(SD) p-value
Age 47.41(13) 46.4(13) 49.2(13)
BMI 43.6(8.0) 42.9(6.8) 44.8(10)
n(%)
Gender
Male 9(20.9) 3(11.1) 6(37.5)
Female 34(79.1) 24(88.9) 10(62.5)
Ethnicity
Irish 30 (69.8) 19 (70.4) 11 (68.8)
Other 4 (9.3) 3 (11.1) 1 (6.3)
Missing 9 (20.9) 5 (18.5) 4 (25.0)
Education level
None 1(2.3) 1(3.7) 0(0)
Primary 2(4.7) 1(3.7) 1(6.3)
Secondary 19(44.2) 12(44.4) 7(43.8)
Third Level 21(48.8) 13(48.1) 8(50.0)
Median (Interquartile range)
Pre-Tutorial Knowledge 2.0 (1.0-2.0) 2.0 (1.0-3.0) 2.0 (1.0-2.0) .414
Pre-Tutorial Self Efficacy 27.0 (31.0-33.0) 32.0(27.0-33.0) 31.0(27.8-33.3) .999
Outcomes
Self-Efficacy Total (n=15) Digital Clinician (n=10) Control (n=5)
Feelings about Injections 5.0 (5.0-5.0) 5.0 (4.0-5.0) .079
Self-Image 5.0 (4.0-5.0) 5.0 (5.0-5.0) .661
Self Confidence 4.5 (4.0-5.0) 5.0 (4.0-5.0) .452
Pain and Skin Reactions 5.0 (5.0-5.0) 5.0 (5.0-5.0) .079
Ease of Use 6.0 (5.0-6.0) 5.0 (5.0-6.0) .215
Satisfaction 5.0 (4.0-5.0) 5.0 (4.0-5.0) .605
Overall Self-Efficacy 10.0 (8.0-10.0) 10.0 (8.2-10.0) .517
Total (n=43) Digital Clinician (n=27) Control (n=16)
Knowledge Raw Score (/12)
Overall Knowledge 10.0 (10.0-11.0) 8.0 (7.0-9.3) <.001
Trust-Distrust Scale Score (/7), Mean(SD)
I am confident of the nurse's knowledge and skills 7.0 (7.0-7.0) 7.0 (7.0-7.0) .176
The nurse cares about you as a person 6.0(4.0-7.0) 7.0 (7.0-7.0) .003
I am(not) suspicious of the nurse's intentions and actions 5.0 (1.5-7.0) 7.0 (6.0-7.0) .002
Overall Trust 7.0 (4.8.-7.0) 7.0 (7.0-7.0) <.001
Consultation Satisfaction
Overall Satisfaction 7.0(6.0-7.0) 7.0 (7.0-7.0) <.001
Usability Digital clinician (n=27), Score (/100)
Interaction Quality 89.6
Interface Quality 81.5
Ease of Use 88.9
Overall 86.7
[illegible]

Would you use/ this Resource in your own time? n(%)

Yes 22(81.5)

No 3(11.1)

Maybe 2(7.4)

15-i) Report demographics associated with digital divide issues

In ehealth trials it is particularly important to report demographics associated with digital divide issues, such as age, education, gender, social-economic status, computer/Internet/ehealth literacy of the participants, if known.

	1	2	3	4	5	
subitem not at all important	○	○	◉	○	○	essential

Clear selection

Does your paper address subitem 15-i? *

Copy and paste relevant sections from the manuscript (include quotes in quotation marks "like this" to indicate direct quotes from your manuscript), or elaborate on this item by providing additional information not in the ms, or briefly explain why the item is not applicable/relevant for your study

Not explored or addressed in the manuscript as the focus was on inter-group analysis rather than inra-group analysis.

16) For each group, number of participants (denominator) included in each analysis and whether the analysis was by original assigned groups

16-i) Report multiple “denominators” and provide definitions

Report multiple “denominators” and provide definitions: Report N’s (and effect sizes) “across a range of study participation [and use] thresholds” [1], e.g., N exposed, N consented, N used more than x times, N used more than y weeks, N participants “used” the intervention/comparator at specific pre-defined time points of interest (in absolute and relative numbers per group). Always clearly define “use” of the intervention.

	1	2	3	4	5	
subitem not at all important	○	○	◉	○	○	essential

Clear selection

Does your paper address subitem 16-i? *

Copy and paste relevant sections from the manuscript (include quotes in quotation marks "like this" to indicate direct quotes from your manuscript), or elaborate on this item by providing additional information not in the ms, or briefly explain why the item is not applicable/relevant for your study

All statistics are of individual groups,not of the whole cohort as is specified at each reference.

16-ii) Primary analysis should be intent-to-treat

Primary analysis should be intent-to-treat, secondary analyses could include comparing only “users”, with the appropriate caveats that this is no longer a randomized sample (see 18-i).

	1	2	3	4	5	
subitem not at all important	○	○	○	◉	○	essential

Clear selection

Does your paper address subitem 16-ii?

Copy and paste relevant sections from the manuscript (include quotes in quotation marks "like this" to indicate direct quotes from your manuscript), or elaborate on this item by providing additional information not in the ms, or briefly explain why the item is not applicable/relevant for your study

This point is addressed by acknowledging the limits of missing data, and through a table 2 which compares those that were lost to follow up with those who were not lost to follow-up, undermining the generalisability of the data, as acknowledged in the manuscript."

"Those with complete data had higher levels of education, female gender, were less trusting of (p=.044) and less satisfied (p=.025) with their healthcare professional than those lost to follow-up- see Supplementary Appendix 3.Table 2. However, the response rates of those in “digital clinician” and the “control” groups at follow-up were similar, as were measures of knowledge, self-efficacy and usability. "

17a) For each primary and secondary outcome, results for each group, and the estimated effect size and its precision (such as 95% confidence interval)

Does your paper address CONSORT subitem 17a? *

Copy and paste relevant sections from the manuscript (include quotes in quotation marks "like this" to indicate direct quotes from your manuscript), or elaborate on this item by providing additional information not in the ms, or briefly explain why the item is not applicable/relevant for your study

See table 1 above.

Your answer must have a minimum of 25 characters.

17a-i) Presentation of process outcomes such as metrics of use and intensity of use

In addition to primary/secondary (clinical) outcomes, the presentation of process outcomes such as metrics of use and intensity of use (dose, exposure) and their operational definitions is critical. This does not only refer to metrics of attrition (13-b) (often a binary variable), but also to more continuous exposure metrics such as "average session length". These must be accompanied by a technical description how a metric like a "session" is defined (e.g., timeout after idle time) [1] (report under item 6a).

	1	2	3	4	5	
subitem not at all important	○	◉	○	○	○	essential

Clear selection

Does your paper address subitem 17a-i?

Copy and paste relevant sections from the manuscript (include quotes in quotation marks "like this" to indicate direct quotes from your manuscript), or elaborate on this item by providing additional information not in the ms, or briefly explain why the item is not applicable/relevant for your study

N/A to this study................

17b) For binary outcomes, presentation of both absolute and relative effect sizes is recommended

Does your paper address CONSORT subitem 17b? *

Copy and paste relevant sections from the manuscript (include quotes in quotation marks "like this" to indicate direct quotes from your manuscript), or elaborate on this item by providing additional information not in the ms, or briefly explain why the item is not applicable/relevant for your study

"Of the 27 participants in the intervention group, 22 (81% of participants affirmed they would use the "digital clinician" for educational purposes in their own time, while 2 (7%) of participants recorded that "maybe" they would. 3 (11%) (3) said they would not. "

18) Results of any other analyses performed, including subgroup analyses and adjusted analyses, distinguishing pre-specified from exploratory

Does your paper address CONSORT subitem 18? *

Copy and paste relevant sections from the manuscript (include quotes in quotation marks "like this" to indicate direct quotes from your manuscript), or elaborate on this item by providing additional information not in the ms, or briefly explain why the item is not applicable/relevant for your study

N/A.................... not performed.

18-i) Subgroup analysis of comparing only users

A subgroup analysis of comparing only users is not uncommon in ehealth trials, but if done, it must be stressed that this is a self-selected sample and no longer an unbiased sample from a randomized trial (see 16-iii).

	1	2	3	4	5	
subitem not at all important	(●)	○	○	○	○	essential

Clear selection

Does your paper address subitem 18-i?

Copy and paste relevant sections from the manuscript (include quotes in quotation marks "like this" to indicate direct quotes from your manuscript), or elaborate on this item by providing additional information not in the ms, or briefly explain why the item is not applicable/relevant for your study

N/A. Once off intervention all users used the intervention as much as each other and it was not self-determined who would use the intervention.

19) All important harms or unintended effects in each group
(for specific guidance see CONSORT for harms)

Does your paper address CONSORT subitem 19? *

Copy and paste relevant sections from the manuscript (include quotes in quotation marks "like this" to indicate direct quotes from your manuscript), or elaborate on this item by providing additional information not in the ms, or briefly explain why the item is not applicable/relevant for your study

Not acknowleged in the paper.

19-i) Include privacy breaches, technical problems

Include privacy breaches, technical problems. This does not only include physical "harm" to participants, but also incidents such as perceived or real privacy breaches [1], technical problems, and other unexpected/unintended incidents. "Unintended effects" also includes unintended positive effects [2].

	1	2	3	4	5	
subitem not at all important	(●)	○	○	○	○	essential

Clear selection

Does your paper address subitem 19-i?

Copy and paste relevant sections from the manuscript (include quotes in quotation marks "like this" to indicate direct quotes from your manuscript), or elaborate on this item by providing additional information not in the ms, or briefly explain why the item is not applicable/relevant for your study

Not detected throughout the study period.

19-ii) Include qualitative feedback from participants or observations from staff/researchers

Include qualitative feedback from participants or observations from staff/researchers, if available, on strengths and shortcomings of the application, especially if they point to unintended/unexpected effects or uses. This includes (if available) reasons for why people did or did not use the application as intended by the developers.

	1	2	3	4	5	
subitem not at all important	○	○	○	◉	○	essential

Clear selection

Does your paper address subitem 19-ii?

Copy and paste relevant sections from the manuscript (include quotes in quotation marks "like this" to indicate direct quotes from your manuscript), or elaborate on this item by providing additional information not in the ms, or briefly explain why the item is not applicable/relevant for your study

"Question: “What do you think about using avatars with automated conversation in a healthcare environment?”
Four responses made reference to a great invention or great possibility for future use. Seven said the idea was good, or very good. Four described it as “okay”. One respondent felt the avatar was “not ideal” and another stated they would “prefer a human”. Two responses were of mixed sentiment-
“I don’t think the real nurse can be replaced but it is great tool for people who live far away to refer to”… “They are helpful but. They are not as good as having a human explaining things to you.”
Question: “What concerns would you have about using avatars with automated conversation like this in the future?”
Twelve responded that they would have no issues using the resource. Two expressed concerns over technical issues such as freezing and audio quality. One expressed concern over language barriers. Four expressed concerns regarding the lack of empathy or possible loss of personal touch. One expressed concern over the ability of the avatar to answer the questions required. 20-25% expressed some form of apprehension.
The prevalence of triggering the wrong conversational stream was not formally measured in the study, however users did not remark on it when providing open ended feedback on usability.
Question: “Any other comments?”
Two responses-
“good to use at home”
“Needle testing could have been shown twice”
"

DISCUSSION

22) Interpretation consistent with results, balancing benefits and harms, and considering other relevant evidence

NPT: In addition, take into account the choice of the comparator, lack of or partial blinding, and unequal expertise of care providers or centers in each group

22-i) Restate study questions and summarize the answers suggested by the data, starting with primary outcomes and process outcomes (use)

Restate study questions and summarize the answers suggested by the data, starting with primary outcomes and process outcomes (use).

	1	2	3	4	5	
subitem not at all important	○	○	◉	○	○	essential

Clear selection

Does your paper address subitem 22-i? *

Copy and paste relevant sections from the manuscript (include quotes in quotation marks "like this" to indicate direct quotes from your manuscript), or elaborate on this item by providing additional information not in the ms, or briefly explain why the item is not applicable/relevant for your study

"Those who received education from the digital clinician were significantly more knowledgeable about their medication and its administration. They tended to have less stress and anxiety associated with using their injections, though this was not significant. Paradoxically, they had less confidence administering injections. They also trusted their education provider significantly less than those in the control group, and were significantly less satisfied with their consultation. This suggests that from a healthcare psychology standpoint, participants' injection-related confidence was more related to who they received their information from, rather than how well informed they were.
While being less satisfied than those in the control group, the intervention group still reported very high levels of trust and satisfaction at levels, with median levels of 7.0/7.0 in both measures. . Participants were extremely positive about the intervention with over 80% of participants expressing that they would use the resource in their own time. If not used clinically, distribution of a "digital clinician" for home use could also add value.
"

22-ii) Highlight unanswered new questions, suggest future research

Highlight unanswered new questions, suggest future research.

	1	2	3	4	5	
subitem not at all important	○	○	○	◉	○	essential

Clear selection

Does your paper address subitem 22-ii?

Copy and paste relevant sections from the manuscript (include quotes in quotation marks "like this" to indicate direct quotes from your manuscript), or elaborate on this item by providing additional information not in the ms, or briefly explain why the item is not applicable/relevant for your study

"Human clinician-patient interactions are not scripted. They're shaped by human factors including rapport and variability. Consultations with the "digital clinician" are more uniform and consistent. This may explain why a human was more successful at ensuring trust and satisfaction, while the "digital clinician" was more effective at ensuring information was provided, tested and retained.
Certain studies have compared physicians versus chatbots, in educational settings, by assessing accuracy. No study has been uncovered that showed that patients themselves were better educated by a chatbot or an LLM than by a specialist clinician. It is one thing to assess what information the chatbot is providing, it is another thing to assess if a patient is receiving, retaining, and trusting that information. Moving forward, only the true benefit of "digital clinicians" can be judged when research examines the risks and benefits of integration on a systemic level.
Standards for new technologies should be established to enable regulation, safety and trust. Bespoke task-specific conversation streams such as this which mimic large language models may be easier to control and regulate while taking advantage of growing trust and recognition in the public domain.
Automation may incorporate trade-offs in patient satisfaction and healthcare system trust, but may offer benefits in comprehensiveness, efficacy and uniformity. Trade-offs could potentially be minimised through research, human oversight and clear physician accountability. The opportunity cost of using these technologies or not, should be considered in terms of productivity, finance, access and workforce and patient health.
"

20) Trial limitations, addressing sources of potential bias, imprecision, and, if relevant, multiplicity of analyses

20-i) Typical limitations in ehealth trials

Typical limitations in ehealth trials: Participants in ehealth trials are rarely blinded. Ehealth trials often look at a multiplicity of outcomes, increasing risk for a Type I error. Discuss biases due to non-use of the intervention/usability issues, biases through informed consent procedures, unexpected events.

	1	2	3	4	5	
subitem not at all important	○	○	○	○	○	essential

Does your paper address subitem 20-i? *

Copy and paste relevant sections from the manuscript (include quotes in quotation marks "like this" to indicate direct quotes from your manuscript), or elaborate on this item by providing additional information not in the ms, or briefly explain why the item is not applicable/relevant for your study

"Limitations of the study include the loss of participants to follow-up questioning due to a global shortage of semaglutide, possibly contributing to some of the non-significant results in the study.. There were significant missing data at 2-weeks follow-up which were omitted from analysis. Table 2 shows the different profile of responders at two-weeks follow-up. Responders tended to have stronger views and were not a reliable representation of the initial cohort, making the conclusions regarding self-efficacy less applicable to the general population.
Non-validated measures of knowledge and adapted measures of usability, trust and consultation satisfaction were used. While the trial design focuses on inter-group analyses, non-validated measures preclude direct comparison of the study cohort with the study population as existing data for the latter does not exist using the same parameters. As a result, non-validated and adapted measures may not correlate as well with other health outcomes such as compliance or the incidence of side effects as established measures.

This feasibility trial, while not measuring traditional health outcomes, would have been strengthened by prospective trial registration, a pre-requisite for all interventional AI trials from the 1st of January 2025 ([38]). Trial registration was initially omitted from the design process. Contributing factors included the novelty of the intervention and the relative sparsity of an established industry proforma at the time. During the trial and data analysis period, further recognition of AI use as a significant healthcare intervention led to the emergence of industry directives, prompting reflection and retrospective trial registration. While not included in this study, further longitudinal research could focus on clinical outcomes such as adherence to therapy or weight loss over longer periods (e.g one year).
"

21) Generalisability (external validity, applicability) of the trial findings

NPT: External validity of the trial findings according to the intervention, comparators, patients, and care providers or centers involved in the trial

21-i) Generalizability to other populations

Generalizability to other populations: In particular, discuss generalizability to a general Internet population, outside of a RCT setting, and general patient population, including applicability of the study results for other organizations

	1	2	3	4	5	
subitem not at all important	○	○	◉	○	○	essential

Clear selection

Does your paper address subitem 21-i?

Copy and paste relevant sections from the manuscript (include quotes in quotation marks "like this" to indicate direct quotes from your manuscript), or elaborate on this item by providing additional information not in the ms, or briefly explain why the item is not applicable/relevant for your study

"There were significant missing data at 2-weeks follow-up which were omitted from analysis. Table 2 shows the different profile of responders at two-weeks follow-up. Responders tended to have stronger views and were not a reliable representation of the initial cohort, making the conclusions regarding self-efficacy less applicable to the general population.
Non-validated measures of knowledge and adapted measures of usability, trust and consultation satisfaction were used. While the trial design focuses on inter-group analyses, non-validated measures preclude direct comparison of the study cohort with the study population as existing data for the latter does not exist using the same parameters. As a result, non-validated and adapted measures may not correlate as well with other health outcomes such as compliance or the incidence of side effects as established measures.
"

21-ii) Discuss if there were elements in the RCT that would be different in a routine application setting

Discuss if there were elements in the RCT that would be different in a routine application setting (e.g., prompts/reminders, more human involvement, training sessions or other co-interventions) and what impact the omission of these elements could have on use, adoption, or outcomes if the intervention is applied outside of a RCT setting.

	1	2	3	4	5	
subitem not at all important	○	○	◉	○	○	essential

Clear selection

Does your paper address subitem 21-ii?

Copy and paste relevant sections from the manuscript (include quotes in quotation marks "like this" to indicate direct quotes from your manuscript), or elaborate on this item by providing additional information not in the ms, or briefly explain why the item is not applicable/relevant for your study

This was not addressed in the manuscript. If the app was to be used routinely, it would be administered in the same setting as in the trial, possibly wthout human supervision to aid with any technical issues that may arise.

OTHER INFORMATION

23) Registration number and name of trial registry

Does your paper address CONSORT subitem 23? *

Copy and paste relevant sections from the manuscript (include quotes in quotation marks "like this" to indicate direct quotes from your manuscript), or elaborate on this item by providing additional information not in the ms, or briefly explain why the item is not applicable/relevant for your study

Your answer

24) Where the full trial protocol can be accessed, if available

Does your paper address CONSORT subitem 24? *

Cite a Multimedia Appendix, other reference, or copy and paste relevant sections from the manuscript (include quotes in quotation marks "like this" to indicate direct quotes from your manuscript), or elaborate on this item by providing additional information not in the ms, or briefly explain why the item is not applicable/relevant for your study

Registration: ISRCTN ISRCTN12382879, https://www.isrctn.com/ISRCTN12382879

25) Sources of funding and other support (such as supply of drugs), role of funders

Does your paper address CONSORT subitem 25? *

Copy and paste relevant sections from the manuscript (include quotes in quotation marks "like this" to indicate direct quotes from your manuscript), or elaborate on this item by providing additional information not in the ms, or briefly explain why the item is not applicable/relevant for your study

No funding was given to this trial.

X27) Conflicts of Interest (not a CONSORT item)

X27-i) State the relation of the study team towards the system being evaluated

In addition to the usual declaration of interests (financial or otherwise), also state the relation of the study team towards the system being evaluated, i.e., state if the authors/evaluators are distinct from or identical with the developers/sponsors of the intervention.

	1	2	3	4	5	
subitem not at all important	○	○	◉	○	○	essential

Clear selection

Does your paper address subitem X27-i?

Copy and paste relevant sections from the manuscript (include quotes in quotation marks "like this" to indicate direct quotes from your manuscript), or elaborate on this item by providing additional information not in the ms, or briefly explain why the item is not applicable/relevant for your study

A third-party app was used, which the research team availed of on a trial basis. No affiliations to the apps used.
"All apps used were from third parties, with no affiliations to the research team."

About the CONSORT EHEALTH checklist

As a result of using this checklist, did you make changes in your manuscript? *

- ○ yes, major changes
- ◉ yes, minor changes
- ○ no

What were the most important changes you made as a result of using this checklist?

Addition of clarity about third party technology app use to clarify regarding potential conflicts of interest.

How much time did you spend on going through the checklist INCLUDING making changes in your manuscript *

Two hours

Your answer must have a minimum of 25 characters.

As a result of using this checklist, do you think your manuscript has improved? *

- (selected) yes
- no
- Other:

Would you like to become involved in the CONSORT EHEALTH group?

This would involve for example becoming involved in participating in a workshop and writing an "Explanation and Elaboration" document

- yes
- (selected) no
- Other:

Clear selection

Any other comments or questions on CONSORT EHEALTH

Thank you.........................

STOP - Save this form as PDF before you click submit

To generate a record that you filled in this form, we recommend to generate a PDF of this page (on a Mac, simply select "print" and then select "print as PDF") before you submit it.

When you submit your (revised) paper to JMIR, please upload the PDF as supplementary file.

Don't worry if some text in the textboxes is cut off, as we still have the complete information in our database. Thank you!

Final step: Click submit !

Click submit so we have your answers in our database!

Submit Clear form

Never submit passwords through Google Forms.

This content is neither created nor endorsed by Google. - Terms of Service - Privacy Policy

Does this form look suspicious? Report

Google Forms
